# A modified sample preparation strategy combined with UHPLC-MS/MS for simultaneous determination of 147 pesticides and related compounds in vegetables and fruits

**DOI:** 10.1016/j.fochx.2026.103506

**Published:** 2026-01-06

**Authors:** Guangyue Wen, Tan Wang, Li Zhao, Wenshuai Si, Hongxia Tang, Maofeng Dong, Yueliang Zhao, Weimin Wang

**Affiliations:** a Pesticide Safety Evaluation Research Center, Institute for Agro-food Standards and Testing Technology, Shanghai Academy of Agricultural Sciences, Shanghai 201106, PR China; b Shanghai Agriculture Technical Extension Service Center, Shanghai 201103, PR China; c School of Public Health, Shanghai Jiao Tong University School of Medicine, Shanghai, China

**Keywords:** Pesticides, Multi-residue, Green extraction, Vegetables and fruits, UHPLC-MS/MS

## Abstract

In this study, a modified approach was described for a simultaneous determination of 147 pesticides and related compounds in vegetables and fruits using ultra-high performance liquid chromatography coupled with triple quadrupole tandem mass spectrometry. The environmentally friendly sample preparation approach consisted of simultaneous extraction of 147 targets with a low dose (2 mL) of ethanol and purification by solid phase extraction. The validation results demonstrated a good linear relationship within the range of 0.002–1 mg/L with a coefficient of determination (R^2^ > 0.99), high sensitivity (0.2–1.0 μg/L for limits of quantification, 0.067–0.33 μg/L for limits of detection and 0.002 mg/kg for method limits of quantification), excellent recoveries (70–119 %), and good precision (RSDs≤19 %). Finally, method comparison and a survey of 54 field samples confirmed that the proposed method was simple, environmentally friendly, and suitable for pesticide multi-residue analysis, which was a good supplement for multi-residue analytical method.

## Highlights

• A modified method was developed for simultaneously analyze 147 target compounds.
• An efficient extraction procedure is proposed using green ethanolic solvent.
• The method is accurate, precise and meets validation requirements by guidelines.
• Method comparation was conducted to assess the analytical performance.
• This method was successfully applied in 4 vegetables and 5 fruits.

## 1 Introduction

Agricultural product safety monitoring plays an irreplaceable role in safeguarding public health (Santos et al., 2025). It is well-known vegetables and fruits are essential foods to provide nutrition, vitamins, minerals and beneficial compounds to humans (Jankowska et al., 2024). But, in order to enhance productivity and control pests and diseases, a variety and a huge number of pesticides are widely used in vegetable and fruit production, bringing concerns about the environment and food safety (Claeys et al., 2011). Particularly for fresh vegetables and fruits, the pesticide residue levels are generally much higher consumption habits of fresh food without cooking and have a higher dietary risk (Tian et al., 2024). So, the establishment of maximum residue limits (MRLs), the development of green pre-treatment technologies and analytical methods, and the dietary risk assessment of pesticides in vegetables and fruits have always been the core work to ensure the manufacturing requirement and safety of food and human health.

Since 1998, Green Analytical Chemistry (GAC) has been constantly developed, discussed, and enhanced (Koel, 2024; Veerendra et al., 2024). The 12 main principles of GAC are increasingly recognized to protect human health and environmental safety during the analytical procedures (Yin et al., 2024). Though it is unlikely to meet all 12 principles, some principles such as reducing reagents and liquid wastes, avoiding derivatives and toxic reagents, controlling energy use, automating and miniaturizing methods are still pursued by analysts, instrument manufacturers, and consumers (Esteve-Turrillas et al., 2024). For the determination of pesticide residues, researchers have also been committed to pursuing low cost, low consumption, and safety to reach the GAC, this requirement is particularly crucial for simultaneously determining hundreds of targets (Akabari et al., 2025). Though benefiting from the rapid development of MS, LC-MS/MS has become the most widely used analytical technique for the determination of pesticides on vegetables and fruits, it is difficult to further improve these techniques (Ibukun et al., 2025; Kottadiyil et al., 2023). Thus, many studies and works put more attention on sample extraction and cleanup (Pallavi et al., 2023). At present, for multiple residues of pesticide monitoring on vegetables and fruits, the development of purification methods is far more than that of extraction technologies (Sadighara et al., 2024). Including dispersive solid-phase extraction (d-SPE), membrane-assisted solvent extraction (MASE), and solid-phase extraction (SPE), these popular cleanup methods were still based on the acetonitrile extraction, which was proposed over 20 years ago (Pan et al., 2025; Santos et al., 2016). So, aiming at the hundreds of analytes, there is still significance in establishing an environmentally friendly and safe sample extraction for vegetables and fruits.

The improvement of sample pretreatment is undoubtedly the main research focus, such as using safer solvents and reaction conditions, preventing waste, etc. (Nguyen et al., 2024). However, for the simultaneous determination of hundreds of targets, the reagent is still an irreconcilable and indispensable contradiction due to the wide variety of pesticides and different sample matrices (Bibi et al., 2022; Carreiró et al., 2024). At present, in most of the research reports and standard analytical methods, acetonitrile has still been the main and most frequently used reagent for sample extraction for over 20 years because of its high extraction efficiency and convenience of operation (Qi et al., 2021). This phenomenon is particularly typical in China's pesticide residue detection standard system. The latest survey showed that all of the national standard analytical methods for pesticides, which could simultaneously determine more than 100 target compounds in vegetables, fruits, cereals, tea, or oil crops, selected acetonitrile as the extraction reagent in China, no matter which pretreatment method was adopted or which determination tool was selected (GAQS, 2008; NHFP, 2021a; Noh et al., 2025). However, in addition to health risks to operators, the toxin of residual waste liquid and the mixture of acetonitrile were another health consideration (Koverga et al., 2017). Therefore, facing the mature traditional pesticide residue analytical methods, how to improve and optimize the existing pretreatment methods is challenging from the perspective of reagent safety.

Therefore, this study was aimed to build an optimal analytical method for simultaneous determination of 147 insecticides, fungicides, plant growth regulators and their related compounds in fresh vegetables and fruits, which were selected based on the recommended pesticides list of the Shanghai Plant Protection Society for preventing and treating main pests and diseases. The main goals of our study were: 1) to modify and optimize the sample pretreatment process by applying ethanol as the extraction reagent instead of traditionally used acetonitrile, and using HLB SPE cartridge for purification; 2) to validate the proposed method in nine vegetables and fruits according to EU guidelines criteria, including four fresh vegetables (cucumber, crown daisy, lettuce and tomato) and five fruits (blueberry, grape, peach, pear, and strawberry); 3) to carry out method comparison research with 2 national standard methods of China; 4) to investigate the application of the proposed method and the pesticide residue levels in the Shanghai region of China using 54 related vegetable and fruit samples.

## 2 Experimental

### 2.1 Chemicals and materials

A 50 mg/L mixed standard solution of 147 pesticides and related compounds (Table S1) was purchased from Beijing Dikma Technology Co., Ltd. (Beijing, China) and stored at −20 ± 2 °C. The working solutions were diluted from the stock solution daily. High Performance Liquid Chromatography (HPLC)-grade ethanol and methanol were purchased from Merck (Darmstadt, Germany), and HPLC-grade formic acid and ammonium formate were obtained from Sigma-Aldrich (St. Louis, MO, USA). Water was purified by a Milli-Q system from Millipore (Billerica, USA). The Hypercarb SPE cartridges (200 mg, 3 mL) were supplied by Thermo Fisher Scientific, Inc. (Shanghai, China). The Waters Oasis® HLB SPE cartridges (60 mg, 3 mL) and Waters Oasis® MAX SPE cartridges (60 mg, 3 mL) were supplied by Waters Corporation (Shanghai, China). The ODS C_18_ SPE cartridges (200 mg, 3 mL) were supplied by Tianjin Bonna Agela Technologies Ltd. (Tianjin, China). The SHIMSEN Styra C_8_ SPE cartridges (200 mg, 3 mL) were supplied by Shimadzu (Shanghai) Global Laboratory Consumables Co., Ltd. (Shanghai, China). The CNW HC-C_18_ SPE cartridges (200 mg, 3 mL), magnesium sulfate (anhydrous MgSO_4_, Analytical Reagents (AR)), sodium chloride (NaCl, AR), trisodium citrate dehydrate (C_6_H_5_Na_3_O_7_·2H_2_O, AR), disodium hydrogen citrate sesquihydrate (C_6_H_6_Na_2_O_7_·1.5H_2_O, AR) were supplied by ANPEL Laboratory Technologies (Shanghai) Inc. (Shanghai, China).

### 2.2 Sample preparation

A thoroughly homogenized sample (2.00 g) was weighed into a 15 mL polytetrafluoroethylene (PTFE) centrifuge tube (tube 1), followed by the addition of 2 mL of ethanol. Then, the mixture was vortexed for 10 min and centrifuged for 5 min at 13750 ×*g*. The entire extraction solution was transferred into another 50 mL centrifuge tube (tube 2). 5 mL of water was added to the remaining samples in tube 1 for a second extraction. The previous vortexing and centrifuging steps were repeated, and the extraction solution was transferred and combined in tube 2. The mixture was then diluted to 20 mL with water, and mixed gently by hand-shaking, and was then ready for purification.

The Oasis HLB cartridges (3 mL/60 mg) were used for the purification. After preconditioned with 3 mL of ethanol and 3 mL of 10 % *v*/v ethanol aqueous solution, 10 mL of extraction solution was loaded on the cartridges and then washing was performed with 3 mL of 10 % v/v ethanol aqueous solution. At last, the targtes were eluted in 1 mL of ethanol, and the eluent was passed through 0.22 μm nylon filter before analysis by UHPLC-MS/MS.

### 2.3 Instrumentation

The qualitative and quantitative determinations were performed on a UHPLC-MS/MS 8060 instrument, equipped with an electrospray ionization (ESI) system (Shimadzu Corp., Tokyo, Japan). A Shim-pack GIST C_18_-AQ HP Column (2.1 mm × 100 mm, 1.9 μm, Shimadzu, Japan) was used to separate the analytes at 40 °C, and the flow rate was set at 0.3 mL/min with an injection volume of 2 μL. Mobile phase A was 0.01 % formic acid with 2 mM ammonium formate in water, and mobile phase B was 0.01 % formic acid with 2 mM ammonium formate in methanol. The gradient elution was as follows: 3 % B (0–1.0 min), increased to 15 % B (1.0–1.5 min); increased to 50 % B (1.5–2.5 min); increased to 70 % B (2.5–18 min); increased to 98 % B (18–23 min); held at 98 % B (23–27 min); ramped to 3 % B (27–27.1 min), and held at 3 % B (27.1–30 min).

The operating conditions for the MS/MS scan were optimized as follows: collision induced dissociation (CID) gas pressure was set at 230 kPa (Ar, 99.999 %); the nebulizing gas and dry gas flow rates were set at 3 L/min and 10.0 L/min respectively (N_2_, 99.9 %); the desolvation line (DL) temperature and heat block temperature were set at 250 °C and 400 °C, respectively. Under the ESI mode, optimized MS/MS transitions of 147 targets were listed in Table S1, and the Shimadzu “LabSolutions® version 5.97” software was used for peak area integration.

### 2.4 Method validation

The established method was evaluated in terms of linearity, limit of detection (LOD), limit of quantification (LOQ), method limit of quantification (mLOQ), matrix effect (ME), accuracy and precision, in accordance with the EU guidelines criteria (European Commission, 2021; Qi et al., 2021). The pesticide standard solutions were prepared in blank matrices at concentrations of 0.002–1 mg/L, which were used to set up a linear curve and estimate the linearity. The LOD and LOQ were determined to be 3 × signal-to-noise ratios, and 10 × signal-to-noise ratios, respectively, and the mLOQ was defined using the lowest spiked concentration (Qi et al., 2021). The ME was calculated as follows: ME (%) = (the slope in matrix/the slope in solvent – 1) × 100 % (Han et al., 2016). If the value was within ±20 %, the ME could be ignored; otherwise, it was necessary to quantify the ME with a matrix standard solution to ensure method accuracy. The positive values and negative values indicated enhancements and suppressions of the analyte signals by matrix interference, respectively (Tang et al., 2023). To evaluate the accuracy and precision of the proposed method, 9 blank vegetable and fruit samples (blueberry, crown daisy, cucumber, grape, lettuce, peach, pear, strawberry and tomato) were spiked with standard solutions at three concentration levels (0.002, 0.01, and 0.1 mg/kg, *n* = 5).

### 2.5 Sample collection

**Fig. 1:**
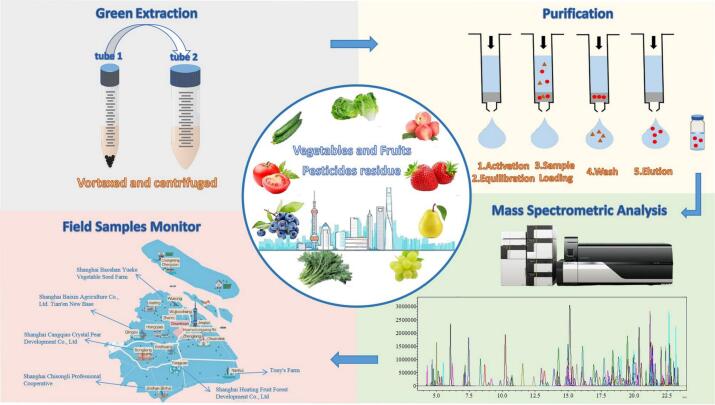
The entire workflow for the green extraction, purification, analysis, and monitor of authentic field samples from Shanghai. (For interpretation of the references to colour in this figure legend, the reader is referred to the web version of this article.)

A total of 54 field vegetable and fruit samples were directly collected from 6 production bases of 5 districts in Shanghai (including Songjiang, Pudong, Qingpu, Jinshan and Fengxian) in 2022, following the guidelines on sampling for pesticide residue analysis (NY/T 789–2004) (MAPR, 2004). In order to ensure the validity of the sample and results, each type of vegetable and fruit was sampled from six different bases and there were six samples for each type. Each sample weighing not less than 3 kg was kept in sterile polythene bags, labeled, and transported to the laboratory within 8 h to avoid deterioration. The samples were serially pulverized by a food processor, numbered, and stored at −20 ± 2 °C, until analysis. The entire workflow for the green extraction, purification, analysis, and monitor of authentic field samples from Shanghai was shown in Fig. 1.

## 3 Results and discussion

### 3.1 Optimization of extraction

Organic solvents, such as acetonitrile, methyl alcohol, acetone, ethyl acetate, etc., are commonly used for pesticide residue extraction (Narayanan et al., 2023; Park et al., 2025; Y. Wang et al., 2022). However, most solvents inevitably have many health and environmental concerns, such as health risk for operators, potential fire hazard, and liquid waste disposal, which becomes the prominent drawback of analytical methods with solvent extraction (Claux et al., 2021). As a Generally Recognized as Safe (GRAS) compound, ethanol was more environmentally friendly due to its low health risk and metabolites (CO_2_ and H_2_O) (Ruangrit et al., 2021). Therefore, ethanol, as a well acknowledged low health risk and low-cost solvent, was investigated for simultaneous determination of pesticides in order to develop a green, safe and reliable method in this work (Calvo-Flores et al., 2018).

**Fig. 2:**
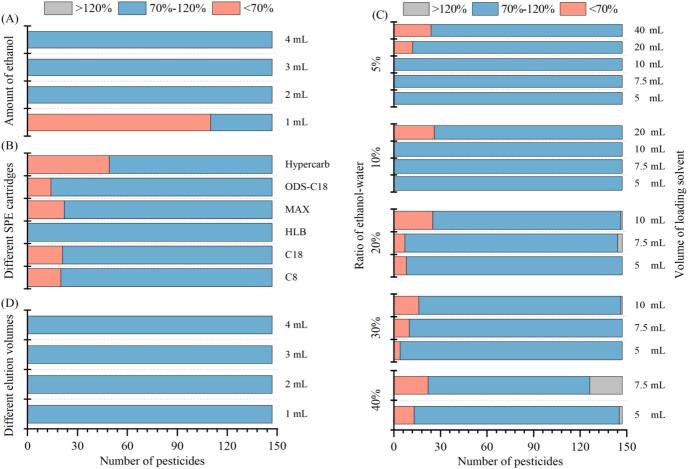
Optimization of extraction with ethanol (A), SPE cartridges (B), volume of loading solvent and ratio of ethanol-water (C), as well as amount of elution volumes (D) for the sample pretreatment (*n* = 3).

First, 2.00 g homogenized blueberry samples were spiked with mixed standard solution at a level of 0.01 mg/kg with 3 parallel samples, and different amounts of ethanol (1 mL, 2 mL, 3 mL and 4 mL) were explored by comparing the fortified recovery to investigate the extraction efficiency. As shown in Fig. 2A, only the recoveries of 37 pesticides and related compounds were higher than 70 %, when 1 mL ethanol was used to extract the target analytes. However, the extraction efficiency of using 2–4 mL ethanol was much more satisfactory, with all the recoveries ranging between 70 and 115 %, and relative standard deviations (RSDs) less than 17 %, meeting the guidelines of EU (European Commission, 2021). These results indicated that 2 mL ethanol was sufficient to provide excellent extraction performance.

### 3.2 Optimization of purification

Ethanol had been proved to be useable as the solvent for multi-residue extraction of pesticides due to its suitable extraction efficiency, but it could not be separated with water by salting out, like acetonitrile, which affected the subsequent determination (Esteve-Turrillas et al., 2024). Hence, the SPE technology made it possible to use ethanol, which was completely miscible with water, for purification by adsorption and elution (Cháfer et al., 2018). After spiking with 0.01 mg/L in the 5 mL blueberry extract solutions (ethanol+water: 1 + 9) and eluted with 3 mL ethanol, the recoveries and RSDs on six commercial SPE cartridges (Hypercarb, ODS-C_18_, MAX, HLB, C_18_, and C_8_ SPE cartridges) were investigated. Fig. 2B showed recoveries of target analytes on the different SPE cartridges were significantly different, and the HLB cartridge provided the best overall recoveries (71–121 %) with RSDs less than 10 %. For the other cartridges, the numbers of analytes with 70–120 % recoveries were 98 for Hypercarb cartridge, 125 for MAX cartridge, 127 for C_8_ cartridge, 126 for C_18_ cartridge, and 133 for ODS-C_18_ cartridge, respectively. The results indicated that HLB cartridge was the best selection among the six tested SPE cartridges to enrich the 147 target analytes from the ethanol/water mixture.

Then, three key indexes of the HLB cartridges (the ethanol percentage in the loading solvent, loading solvent volume, and the elution reagent) were specifically investigated by checking fortified recoveries in order to improve the method performance. Blueberry extract solutions spiked with three replicates of 0.01 mg/L mixed standard solution were used to evaluate the aqueous ethanol percentage of 5 %, 10 %, 20 %, 30 %, 40 %, and the loading solution of 5 mL, 7.5 mL, 10 mL, 20 mL, and 40 mL. Fig. 2C showed that the aqueous ethanol percentage and loading volumes highly influenced the performance of HLB cartridge. The higher aqueous ethanol percentage and larger loading volumes caused penetration through the HLB cartridges, consistent with existing papers (Shahsavani & Fakhari, 2024; T. Wang et al., 2024). The results apparently showed only when aqueous ethanol percentage and the loading volume were not exceeded 10 % and 10 mL, all the recoveries (71–115 %) were acceptable. For example, when aqueous ethanol percentage was 10 % with 20 mL loading solvent, recoveries of 26 target analytes (amisulbrom, cyetpyrafen, ivermectin, methoprene, novaluron, *etc*) were 53–69 %, but the recoveries of 26 analytes increased to 72–101 % when the loading volume was reduced to 10 mL with the same aqueous ethanol percentage. Therefore, to ensure operability and recovery, the aqueous ethanol percentage should be kept 10 % by adding water to 20 mL, while the loading volume should be less than 10 mL during the SPE purification process.

For elution reagent, ethanol was ulteriorly evaluated and optimized to avoid using of other highly toxic solvents. The effect of different elution volumes on the recovery was tested to explore the minimum effective elution volume. After loading 10 mL blueberry extract solution (10 % aqueous ethanol, and spiked with 0.01 mg/L mixed standard solution, *n* = 3), 1 mL, 2 mL, 3 mL, and 4 mL ethanol were sequentially used to elute the analytes from the HLB cartridge. As shown in Fig. 2D, the recoveries ranged from 72 % to 107 % with RSDs of 0–11 % when the elution volumes of 1–4 mL were used. Therefore, the minimum elution volume of 1 mL ethanol was chosen to elute 147 target analytes.

In total, the whole components of the sample pretreatment were optimized and obtained, including 2 mL ethanol for extraction, and the HLB cartridge for purification with aqueous ethanol percentage of 10 %, loading volume of 10 mL and ethanol elution volume of 1 mL.

### 3.3 Method validation

Nine frequently consumed vegetables and fruits including four fresh vegetables (cucumber, crown daisy, lettuce, and tomato) and five fruits (blueberry, grape, peach, pear, and strawberry) were chosen as the validated matrices to evaluate the performance of the proposed method following EU guideline criteria for the validation of analytical method (European Commission, 2021).

In the calibration range of 0.002–0.1 mg/L (0.002, 0.005, 0.01, 0.05, and 0.1 mg/L), the linear equations of 147 analytes showed good linearity, with the correlation coefficients (R^2^) exceeding 0.99. The LODs and LOQs of analytes were ranged from 0.067 to 0.33 μg/L and 0.2–1 μg/L, respectively, and the mLOQs were all 0.002 mg/kg, which were much lower than the lowest existing MRLs (0.02 mg/kg) for this nine crops of the target pesticides in China, demonstrating excellent sensitivity of the proposed method (NHFP, 2021b).

In Table S2, the MEs were significantly different between the targets and matrices, ranging from −81-317 %. The MEs were divided into three groups following the evaluation criteria (below−20 %, between ±20 %, and above 20 %). Among them, 12 % of targets had negative matrix effects ranging from −81−−21 %, and the lowest value was −81 % for cyflumetofen in the crown daisy matrix. 72 % of targets had no obvious matrix effect with the values in ranging of ±20 %. The remaining 17 % had positive matrix effects in the range of 21–317 %, with the highest value of 317 % for *N*-demethyl-175-J in tomato matrix. As 29 % of the target analytes had significant matrix effects, the matrix-matched calibration standard was still indispensable to ensure accurate quantification to compensate for matrix effects. Table S2 also showed the recoveries of 147 targets in 9 matrices were 70–119 % at the 0.002 mg/kg spiked level, 71–110 % at the 0.01 mg/kg spiked level, and 72–114 % at the 0.1 mg/kg spiked level. For RSDs, the values were 1–19 %, 1–18 %, and 1–17 % for the three spiking levels, respectively. These results showed the accuracy and precision of the method were acceptable and stable, in accordance with the EU guideline criteria for validation of analytical method (European Commission, 2021). The entire workflow was outlined in the flowchart shown in Fig. 1.

### 3.4 Method comparison

To further evaluate the performance of the proposed method, two National Food Safety Standards of China for pesticide multi-residue analysis (GB 23200.121 (method 1) and GB 20769 (method 2) (GAQS, 2008; NHFP, 2021a), which separately adopted d-SPE and SPE, were selected and compared by the sample pretreatment, spike recovery, ME, solvent consumption, etc. As summarized in Table 1, the sample pretreatment of method 1 was the simplest due to the application of d-SPE, but there were many cumbersome weighing steps, such as 10 g sample, 4 g anhydrous MgSO_4_, 1 g NaCl, 1 g C_6_H_5_Na_3_O_7_·2H_2_O, and 0.5 g C_6_H_6_Na_2_O_7_·1.5H_2_O, 150 mg/mL anhydrous MgSO_4_, and 25 mg/mL PSA; For method 2, the SPE step (1 mL loading solvent and 31 mL for elution) and two rotary evaporation steps (20 mL and 31 mL) took a relatively long time. Compared with the two national standard methods, the current method (method 3) was more suitable and more acceptable with only one weighing step and SPE (10 mL loading solvent and 1 mL for elution) procedure without rotary evaporation. Moreover, in terms of solvent consumption, the current method 3 (only 7 mL ethanol in the whole sample pretreatment) was much more environmentally friendly and safer, compared with 10 mL acetonitrile in method 1, 67 mL acetonitrile and 9 mL toluene in method 2. Hence, the low-volume application of ethanol greatly enhanced the personal exposure protection, reduced health risks, and significantly simplified the liquid waste disposal, which was more aligned with the trend of green chemistry development.

**Table 1:** A comparison of the current method with standard methods for the analysis of multiple pesticide residues in vegetables and fruits.

Method description	Method 1 (GB 23200.121)	Method 2 (GB 20769)	The current method
Brief description	The sample was extracted with acetonitrile, and the extract was purified by d-SPE. LC-MS/MS was used for detection, and the external standard method was used for quantification.	The sample was extracted with acetonitrile, and the extract was purified by SPE. LC-MS/MS was used for detection, and the external standard method was used for quantification.	The sample was extracted with ethanol, and the extract was purified by SPE. LC-MS/MS was used for detection, and the external standard method was used for quantification.
Extraction reagents	10 mL of acetonitrile, with 4 g anhydrous MgSO_4_, 1 g NaCl, 1 g C_6_H_5_Na_3_O_7_·2H_2_O, and 0.5 g C_6_H_6_Na_2_O_7_·1.5H_2_O	40 mL of acetonitrile, with 5 g NaCl	2 mL of ethanol and 5 mL of water
Cleanup sorbent	anhydrous MgSO_4_, PSA	Sep-Pak Vac	Oasis HLB
Consumption of organic solvents (mL)	10	76	7
Sample preparation time (min)	20	120	50
%pesticides with 70–120 % recovery at 100 ng/g	100 (147/147)	96 (141/147)	100 (147/147)
% pesticides with matrix effect >20 %	1 (cucumber), 11 (strawberry)	1 (cucumber), 10 (strawberry)	1 (cucumber), 10 (strawberry)
% pesticides with matrix effect between ±20 %	41 (cucumber), 85 (strawberry)	0 (cucumber), 85 (strawberry)	74 (cucumber), 86 (strawberry)
% pesticides with matrix effect <20 %	58 (cucumber), 4 (strawberry)	99 (cucumber), 5 (strawberry)	25 (cucumber), 4 (strawberry)

**Fig. 3:**
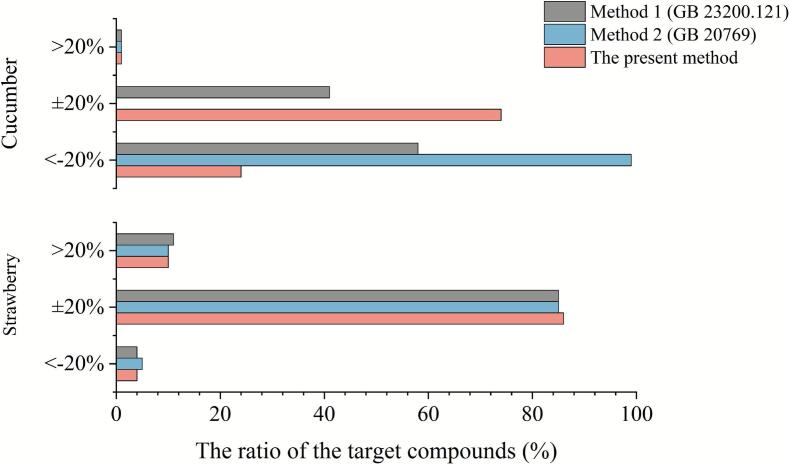
The matrix effects (MEs, %) of 147 pesticides and related compounds in typical vegetables (crowndaisy) and fruits (peach) determined by the proposed method and two of the National Food Safety Standards of China for pesticide multi-residue analysis (GB 23200.121 (method 1) and GB 20769 (method 2).

In terms of precision, accuracy, sensitivity and purification effect, method 3 also displayed acceptable results. Spiked at 0.1 mg/kg in cucumber or strawberry samples, Table 1 showed all the targets in method 3 and method 1 obtained satisfied recoveries of 70–120 % for all the 147 targets. For method 2, 96 % of the target compounds (141/147) obtained acceptable recoveries ranging from 70 % to 120 %, but the recovery results for the remaining 6 compounds were unacceptable (carboxin, spirotetramat-enol, albendazole, fludioxonil, pyrethrin I and methoprene). For sensitivity, the mLOQ of method 1 was 0.01 mg/kg, which was much higher than the mLOQ (0.002 mg/kg) verified in method 3. For MEs, as shown in Table 1 and Fig. 3, 74 % of analytes in cucumber and 86 % in strawberry using method 3 had the values within the requirement of ±20 %, indicating higher purification efficiency than the other two methods. Based on these results, the proposed method had excellent performance, which was expected to reduce the use of reagents, improve sensitivity and purification efficiency, and ensure operability.

### 3.5 Pesticide detection in samples

**Fig. 4:**
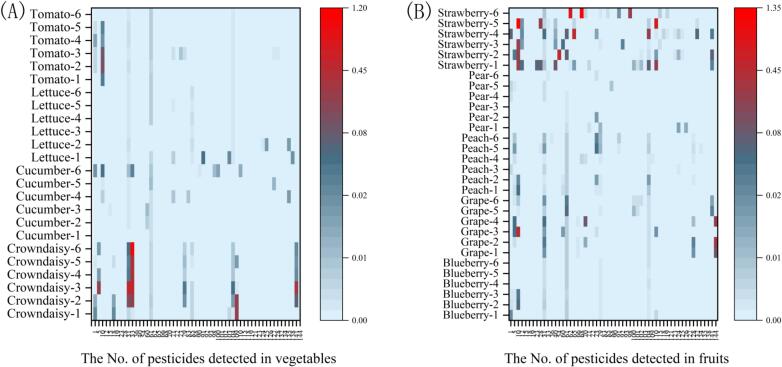
Survey of the pesticides residue levels in authentic field vegetables (A) and fruits (B).

As part of our research goal, pesticide residues in 54 field samples from Shanghai production bases were analyzed, in order to reduce the influence of transportation and preservation and better reflect the pesticide residues in the locally grown produce. The preliminary data would be applied to evaluate the proposed method and provide some food safety guidance to government and local consumers. As presented in Fig. 4, the results showed that pesticide residue detection rate was 100 % (54 out of 54), and a total of 56 pesticides were detected across all samples including 17 insecticides, 38 bactericides, and 1 plant growth regulator, with the residue level in the range of 0.002–1.42 mg/kg. Among them, the highest residue level was 1.26 mg/kg of dimethomorph in crown daisy for vegetable samples, and 1.42 mg/kg of boscalid in strawberry for fruit samples.

According to GB 2763–2021 and GB 2763.1–2022 in China (NHFP, 2021b, NHFP, 2022), although there were 242 MRLs for 56 pesticides detected on the 9 selected vegetables and fruits, almost 70 % MRLs were set on the conventional crops (40 in cucumber, 35 in grape, 29 in pear, 28 in strawberry and 36 in tomato). For the small crops, there were only 74 MRLs (17 in blueberry, 11 in crown daisy, 27 in peach, and 19 in lettuce), much less than the total number of 168 on the conventional crops. Among all the 443 detected data within the 9 commodities, 64 % (283/443) had their homologous MRLs, and all those residues were below the existing MRLs. In the remaining 7 % (31/443) pesticides detected, there were 11 pesticides registered on related crops without MRLs in China, including grape (ethirimol, fluazinam, and lufenuron), peach (forchlorfenuron, paclobutrazol, and pyridaben), crown daisy (dimethomorph), cucumber (clothianidin) and strawberry (bupirimate, cyetpyrafen, ethirimol, fluazinam, and prochloraz). Nevertheless, in the other remaining 29 % (129/443), 21 pesticides were not registered in the relative crops, such as ethirimol in blueberry, grape, peach, tomato, and crown daisy. In those pesticides, the small crops were the main concern, the proportion of data without MRL or registration was 78 % (100/129), including 17 for blueberry, 49 for crown daisy, 17 for peach and 17 for lettuce, compared with the proportion of 21 % (29/129) for the other five crops. As for pesticide registration on small crops, the demand for disease pest control and the requirements of the pesticide management regulations seemed not to be equivalent. Therefore, to control the plant diseases and insect pests on small crops, speeding up the pesticide registration was the main pathway for the problem, particularly for the crops with high consumption, such as leaf lettuce, which would also provide sufficient data for establishing MRLs on crops in China.

Meanwhile, consistent with existing literatures, the problem of the pesticides multi-residues in a single sample was also worth attention (Huertas-Pérez et al., 2024). For example, in 6 strawberry samples, a total of 31 pesticides had been detected, wherein, 2 samples were detected with more than 15 pesticides, and 4 samples were detected with 10–15 pesticides. During the crop growing process, multiple pesticides were inevitably used to control various diseases and pests. However, even though the levels of pesticide residues were lower than the MRLs, the amounts of pesticides or mixed toxicity may bring potential risk, which was indeed an important challenge in the evaluation of dietary safety (Park et al., 2025).

## 4 Conclusion

In this work, we established and validated a modified environmentally friendly methodology based on ethanol extraction, clean-up using HLB cartridges and analysis of UHPLC-MS/MS. Compared to the existing reports and standards, this work proposed a safe and green sample pretreatment for over 100 pesticides, which would be a good supplement for the traditional acetonitrile extraction method. As only 7 mL of ethanol was applied in the sample treatment, this procedure significantly reduced the potential hazardous exposure to the operator and simplified the liquid waste disposal process. The current method complied with the expectation of GAC and exhibits satisfactory performance in terms of linearity, sensitivity, accuracy and precision compared to other standard methods. Furthermore, it had been successfully applied to the determination of 147 pesticides and related compounds in 54 real vegetable and fruit samples collected from the production bases in Shanghai. The present study could provide technical support for the further control and dietary assessment research of pesticides in vegetables and fruits and could also be considered as an alternative to the traditional acetonitrile based analytical methods.

## CRediT authorship contribution statement

**Guangyue Wen:** Writing – original draft, Methodology, Investigation. **Tan Wang:** Writing – original draft, Methodology, Investigation. **Li Zhao:** Project administration, Conceptualization. **Wenshuai Si:** Data curation. **Hongxia Tang:** Project administration, Investigation. **Maofeng Dong:** Writing – review & editing, Supervision, Project administration, Investigation. **Yueliang Zhao:** Writing – review & editing. **Weimin Wang:** Supervision, Resources, Conceptualization.

## Declaration of competing interest

The authors declare that they have no known competing financial interests or personal relationships that could have influenced the work reported in this paper.

## Data Availability

No data was used for the research described in the article.

## Appendix A Supplementary data

Supplementary material Image 1
