# Supplementary material for: A modified sample preparation strategy combined with UHPLC-MS/MS for simultaneous determination of 147 pesticides and related compounds in vegetables and fruits
Source: Food Chem X. 2026 Jan 6;33:103506. doi: 10.1016/j.fochx.2026.103506 (PMC12816860; doi:10.1016/j.fochx.2026.103506)
Supplement: Supplementary file 1 — Supplementary material [file mmc1.docx]

**Supplementary Materials For:**

**A modified sample preparation strategy combined with UHPLC-MS/MS for simultaneous determination of 147 pesticides and related compounds in vegetables and fruits**

Guangyue Wen^a,1^, Tan Wang^a,1^, Li Zhao^b^, Wenshuai Si^a^, Hongxia Tang^a,*^ Maofeng Dong^a,*^, Yueliang Zhao^c^, Weimin Wang^a^

^a^ Pesticide Safety Evaluation Research Center, Institute for Agro-food Standards and Testing Technology, Shanghai Academy of Agricultural Sciences, Shanghai 201106, *PR China*.

^b^ Shanghai Agriculture Technical Extension Service Center, Shanghai, 201103, PR China.

^c^ School of Public Health, Shanghai Jiao Tong University School of Medicine, Shanghai, China.

^1^Guangyue Wen and Tan Wang contributed equally to the work

To whom correspondence should be addressed:

Mrs. Hongxia Tang, [lobsterjane@163.com](mailto:lobsterjane@163.com)

Mr. Maofeng Dong, [dmf385@163.com](mailto:dmf385@163.com)

**Table S1** List of selected pesticides and related compounds for analysis, molecular formula, CAS, retention time, and MS/MS condition.

| No. | Pesticide | Molecular formula | CAS | Retention time (min) | Precursor ion/Product ion  (Q/q, *m/z*) | Q1 Pre Bias (V) | CE (V) | Q3 Pre Bias (V) |
| --- | --- | --- | --- | --- | --- | --- | --- | --- |
| 1 | Acetamiprid | C_10_H_11_ClN_4_ | 135410-20-7 | 5.017 | 223.10>126.05/ | -30 | -22 | -30 |
|  |  |  |  |  | 223.10>56.10 | -30 | -15 | -23 |
| 2 | Albendazole | C_12_H_15_N_3_O_2_S | 54965-21-8 | 13.187 | 266.00>234.00/ | -12 | -30 | -23 |
|  |  |  |  |  | 266.00>191.00 | -12 | -25 | -30 |
| 3 | Ametoctradin | C_15_H_25_N_5_ | 865318-97-4 | 21.131 | 276.20>176.10/ | -10 | -35 | -20 |
|  |  |  |  |  | 276.20>149.00 | -10 | -35 | -17 |
| 4 | Amisulbrom | C_13_H_13_BrFN_5_O_4_S_2_ | 348635-87-0 | 22.103 | 466.00>227.00/ | -10 | -19 | -13 |
|  |  |  |  |  | 466.00>148.00 | -15 | -40 | -27 |
| 5 | Azoxystrobin | C_22_H_17_N_3_O_5_ | 131860-33-8 | 14.089 | 404.10>372.05/ | -30 | -25 | -26 |
|  |  |  |  |  | 404.10>329.00 | -30 | -28 | -23 |
| 6 | Benalaxyl | C_20_H_23_NO_3_ | 71626-11-4 | 19.818 | 326.20>148.20/ | -16 | -13 | -15 |
|  |  |  |  |  | 326.20>294.10 | -16 | -15 | -20 |
| 7 | Benzovindiflupyr | C_18_H_15_Cl_2_F_2_N_3_O | 1072957-71-1 | 19.969 | 398.00>342.00/ | -15 | -18 | -23 |
|  |  |  |  |  | 398.00>378.00 | -15 | -14 | -26 |
| 8 | Benzoximate | C_18_H_18_ClNO_5_ | 29104-30-1 | 21.533 | 364.10>199.00/ | -13 | -12 | -23 |
|  |  |  |  |  | 364.10>105.00 | -27 | -26 | -21 |
| 9 | Bitertanol | C_20_H_23_N_3_O_2_ | 55179-31-2 | 20.795 | 338.20>269.15/ | -17 | -9 | -29 |
|  |  |  |  |  | 338.20>99.10 | -17 | -15 | -18 |
| 10 | Boscalid | C_18_H_12_Cl_2_N_2_O | 188425-85-6 | 14.084 | 343.00>307.10/ | -12 | -18 | -30 |
|  |  |  |  |  | 343.00>271.10 | -12 | -30 | -26 |
| 11 | Bromuconazole | C_13_H_12_BrCl_2_N_3_O | 116255-48-2 | 15.057 | 377.90>158.90/ | -19 | -28 | -30 |
|  |  |  |  |  | 377.90>70.00 | -19 | -23 | -30 |
| 12 | Bupirimate | C_13_H_24_N_4_O_3_S | 41483-43-6 | 18.299 | 317.10>108.00/ | -30 | -26 | -19 |
|  |  |  |  |  | 317.10>210.20 | -30 | -23 | -22 |
| 13 | Buprofezin | C_16_H_23_N_3_OS | 69327-76-0 | 22.522 | 306.10>116.10/ | -30 | -23 | -12 |
|  |  |  |  |  | 306.10>201.10 | -30 | -20 | -22 |
| 14 | Carboxin | C_12_H_13_NO_2_S | 5234-68-4 | 8.576 | 236.05>143.00/ | -10 | -22 | -16 |
|  |  |  |  |  | 236.05>87.00 | -10 | -23 | -19 |
| 15 | Chlorantraniliprole | C_18_H_14_BrCl_2_N_5_O_2_ | 500008-45-7 | 12.239 | 484.00>452.90/ | -24 | -19 | -30 |
|  |  |  |  |  | 484.00>285.85 | -24 | -16 | -30 |
| 16 | Chlorfluazuron | C_20_H_9_Cl_3_F_5_N_3_O_3_ | 71422-67-8 | 23.673 | 540.00>382.90/ | -26 | -21 | -27 |
|  |  |  |  |  | 540.00>158.00 | -26 | -20 | -30 |
| 17 | Chromafenozide | C_24_H_30_N_2_O_3_ | 143807-66-3 | 17.195 | 395.30>175.10/ | -14 | -40 | -20 |
|  |  |  |  |  | 395.30>339.15 | -15 | -7 | -19 |
| 18 | Clothianidin | C_6_H_8_ClN_5_O_2_S | 210880-92-5 | 4.781 | 250.00>169.11/ | -29 | -12 | -17 |
|  |  |  |  |  | 250.00>132.00 | -29 | -14 | -24 |
| 19 | Coumoxystrobin | C_26_H_28_O_6_ | 850881-70-8 | 22.949 | 437.10>205.10/ | -12 | -10 | -16 |
|  |  |  |  |  | 437.10>145.10 | -12 | -35 | -17 |
| 20 | Cyantraniliprole | C_19_H_14_BrClN_6_O_2_ | 736994-63-1 | 8.877 | 475.00>286.00/ | -11 | -19 | -22 |
|  |  |  |  |  | 475.00>444.00 | -17 | -19 | -24 |
| 21 | Cyazofamid | C_13_H_13_ClN_4_O_2_S | 120116-88-3 | 17.525 | 325.00>108.05/ | -11 | -12 | -21 |
|  |  |  |  |  | 325.00>261.10 | -24 | -11 | -30 |
| 22 | CCIM (4-Chlor-2-cyano-5-(4-methylphenyl)  imidazol) | C_11_H_8_ClN_3_ | 120118-14-1 | 14.806 | 216.00>179.10/ | 10 | 31 | 16 |
|  |  |  |  |  | 216.00>180.15 | 15 | 25 | 17 |
| 23 | Cyetpyrafen | C_24_H_31_N_3_O_2_ | 1253429-01-4 | 23.624 | 394.50>310.15/ | -18 | -25 | -10 |
|  |  |  |  |  | 394.50>254.10 | -18 | -31 | -10 |
| 24 | Cyflufenamid | C_20_H_17_F_5_N_2_O_2_ | 180409-60-3 | 21.140 | 413.20>295.05/ | -20 | -10 | -30 |
|  |  |  |  |  | 413.20>203.00 | -20 | -30 | -20 |
| 25 | Cyflumetofen | C_24_H_24_F_3_NO_4_ | 400882-07-7 | 22.323 | 465.20>173.00/ | -14 | -24 | -18 |
|  |  |  |  |  | 465.20>145.00 | -12 | -14 | -25 |
| 26 | Cymoxanil | C_7_H_10_N_4_O_3_ | 57966-95-7 | 5.388 | 199.10>128.10/ | -21 | -8 | -25 |
|  |  |  |  |  | 199.10>111.10 | -21 | -18 | -21 |
| 27 | Cyproconazole | C_15_H_18_ClN_3_O | 94361-06-5 | 15.242 | 292.10>70.05/ | -30 | -20 | -27 |
|  |  |  |  |  | 292.10>125.05 | -30 | -30 | -22 |
| 28 | Cyprodinil | C_14_H_15_N_3_ | 121552-61-2 | 19.450 | 226.10>93.10/ | -30 | -34 | -16 |
|  |  |  |  |  | 226.10>9108.10 | -30 | -27 | -19 |
| 29 | Diclobutrazol | C_15_H_19_Cl_2_N_3_O | 75736-33-3 | 18.383 | 328.00>70.00/ | -12 | -21 | -15 |
|  |  |  |  |  | 328.00>70.00 | -12 | -22 | -15 |
| 30 | Diethofencarb | C_14_H_21_NO_4_ | 87130-20-9 | 12.792 | 268.10>226.10/ | -30 | -15 | -24 |
|  |  |  |  |  | 268.10>180.10 | -30 | -25 | -19 |
| 31 | Difenoconazole | C_19_H_17_Cl_2_N_3_O_3_ | 119446-68-3 | 21.296 | 406.10>251.01/ | -30 | -25 | -27 |
|  |  |  |  |  | 406.10>337.05 | -30 | -17 | -24 |
| 32 | Diflubenzuron | C_14_H_9_ClF_2_N_2_O_2_ | 35367-38-5 | 17.872 | 311.00>158.00/ | -11 | -15 | -30 |
|  |  |  |  |  | 311.00>141.20 | -11 | -24 | -11 |
| 33 | Dimethomorph | C_21_H_22_ClNO_4_ | 110488-70-5 | 14.749 | 388.10>301.00/ | -19 | -30 | -21 |
|  |  |  |  |  | 388.10>165.05 | -19 | -25 | -30 |
| 34 | Dimoxystrobin | C_19_H_22_N_2_O_3_ | 149961-52-4 | 18.776 | 327.00>205.05/ | -30 | -15 | -30 |
|  |  |  |  |  | 327.00>116.00 | -30 | -35 | -30 |
| 35 | Diniconazole | C_15_H_17_Cl_2_N_3_O | 83657-24-3 | 20.781 | 326.10>70.00/ | -12 | -24 | -15 |
|  |  |  |  |  | 326.10>159.00 | -12 | -27 | -18 |
| 36 | Emamectinbenzoate | C_56_H_81_NO_15_ | 137512-74-4 | 22.607 | 886.50>158.10/ | -40 | -25 | -17 |
|  |  |  |  |  | 886.50>82.10 | -40 | -55 | -15 |
| 37 | Enestroburin | C_22_H_22_ClNO_4_ | 238410-11-2 | 22.626 | 400.10>178.00/ | -19 | -25 | -14 |
|  |  |  |  |  | 400.10>137.00 | -15 | -15 | -16 |
| 38 | Epoxiconazole | C_17_H_13_ClFN_3_O | 133855-98-8 | 16.955 | 330.10>121.20/ | -12 | -20 | -26 |
|  |  |  |  |  | 330.10>101.00 | -12 | -43 | -21 |
| 39 | Ethiprole | C_13_H_9_Cl_2_F_3_N_4_OS | 181587-01-9 | 13.202 | 397.00>255.00/ | -20 | -45 | -26 |
|  |  |  |  |  | 397.00>351.00 | -20 | -15 | -24 |
| 40 | Ethirimol | C_11_H_19_N_3_O | 23947-60-6 | 8.686 | 210.20>140.10/ | -13 | -22 | -25 |
|  |  |  |  |  | 210.20>98.05 | -13 | -26 | -16 |
| 41 | Famoxadone | C_22_H_18_N_2_O_4_ | 131807-57-3 | 20.810 | 392.00>331.00/ | -11 | -12 | -25 |
|  |  |  |  |  | 392.00>238.00 | -13 | -20 | -10 |
| 42 | Fenamidone | C_17_H_17_N_3_OS | 161326-34-7 | 13.970 | 312.10>236.10/ | -11 | -15 | -24 |
|  |  |  |  |  | 312.10>92.05 | -11 | -24 | -16 |
| 43 | Fenarimol | C_17_H_12_Cl_2_N_2_O | 60168-88-9 | 16.620 | 331.00>268.10/ | -16 | -22 | -28 |
|  |  |  |  |  | 331.00>259.10 | -17 | -26 | -26 |
| 44 | Fenbuconazole | C_19_H_17_ClN_4_ | 114369-43-6 | 17.485 | 336.90>125.05/ | -26 | -27 | -25 |
|  |  |  |  |  | 336.90>70.00 | -26 | -20 | -28 |
| 45 | Fenhexamid | C_14_H_17_Cl_2_NO_2_ | 126833-17-8 | 15.960 | 301.90>97.10/ | -15 | -23 | -19 |
|  |  |  |  |  | 301.90>55.05 | -15 | -40 | -22 |
| 46 | Fenoxanil | C_15_H_18_Cl_2_N_2_O_2_ | 115852-48-7 | 17.568 | 329.10>302.10/ | -17 | -12 | -30 |
|  |  |  |  |  | 329.10>86.10 | -17 | -22 | -15 |
| 47 | Fenpropidin | C_19_H_31_N | 67306-00-7 | 11.442 | 274.10>147.10/ | -30 | -35 | -30 |
|  |  |  |  |  | 274.10>117.20 | -14 | -53 | -21 |
| 48 | Fenpropimorph | C_20_H_33_NO | 67564-91-4 | 16.624 | 304.20>147.20/ | -30 | -24 | -27 |
|  |  |  |  |  | 304.20>119.10 | -30 | -30 | -22 |
| 49 | Fenpyrazamine | C_17_H_21_N_3_O_2_S | 473798-59-3 | 15.881 | 332.00>304.00/ | -16 | -13 | -17 |
|  |  |  |  |  | 332.00>272.00 | -12 | -13 | -21 |
| 50 | Fipronil | C_12_H_4_C_l2_F_6_N_4_OS | 120068-37-3 | 18.090 | 435.00>330.00/ | 10 | 16 | 21 |
|  |  |  |  |  | 435.00>250.00 | 10 | 28 | 24 |
| 51 | Fipronil-desulfinyl | C_12_H_4_C_l2_F_6_N_4_ | 205650-65-3 | 17.507 | 387.00>351.00/ | 14 | 17 | 23 |
|  |  |  |  |  | 387.00>282.00 | 14 | 32 | 17 |
| 52 | Fipronil-sulfide | C_12_H_4_Cl_2_F_6_N_4_S | 120067-83-6 | 19.262 | 419.00>262.00/ | 16 | 29 | 16 |
|  |  |  |  |  | 419.00>383.00 | 12 | 13 | 17 |
| 53 | Fipronil-sulfone | C_12_H_4_Cl_2_F_6_N_4_O_2_S | 120068-36-2 | 20.220 | 451.00>415.00/ | 17 | 17 | 19 |
|  |  |  |  |  | 451.00>282.00 | 17 | 27 | 18 |
| 54 | Fluazinam | C_13_H_4_Cl_2_F_6_N_4_O4 | 79622-59-6 | 22.600 | 463.00>416.00/ | 22 | 20 | 13 |
|  |  |  |  |  | 463.00>398.00 | 13 | 17 | 17 |
| 55 | Flubendiamide | C_23_H_22_F_7_IN_2_O_4_S | 272451-65-7 | 19.766 | 681.00>254.05/ | 32 | 26 | 27 |
|  |  |  |  |  | 681.00>2573.80 | 32 | 15 | 28 |
| 56 | Fludioxonil | C_12_H_6_F_2_N_2_O_2_ | 131341-86-1 | 14.221 | 266.10>229.00/ | -10 | -14 | -18 |
|  |  |  |  |  | 266.10>158.00 | -10 | -46 | -19 |
| 57 | Flufenoxuron | C_21_H_11_ClF_6_N_2_O_3_ | 101463-69-8 | 23.275 | 489.00>158.10/ | -11 | -20 | -12 |
|  |  |  |  |  | 489.00>141.20 | -11 | -39 | -17 |
| 58 | Flumetralin | C_16_H_12_ClF_4_N_3_O_4_ | 62924-70-3 | 23.713 | 422.10>107.10/ | -22 | -54 | -22 |
|  |  |  |  |  | 422.10>143.00 | -15 | -47 | -27 |
| 59 | Flumorph | C_21_H_22_FNO_4_ | 211867-47-9 | 11.444 | 372.10>285.05/ | -17 | -15 | -29 |
|  |  |  |  |  | 372.10>165.15 | -17 | -23 | -28 |
| 60 | Fluopicolide | C_14_H_8_Cl_3_F_3_N_2_O | 239110-15-7 | 14.774 | 382.90>172.95/ | -17 | -22 | -30 |
|  |  |  |  |  | 382.90>145.00 | -17 | -47 | -24 |
| 61 | Fluopyram | C_16_H_11_ClF_6_N_2_O | 658066-35-4 | 15.901 | 397.00>172.95/ | -28 | -20 | -18 |
|  |  |  |  |  | 397.00>207.90 | -28 | -15 | -22 |
| 62 | Flusilazole | C_16_H_15_F_2_N_3_Si | 85509-19-9 | 17.831 | 316.10>247.10/ | -30 | -18 | -27 |
|  |  |  |  |  | 316.10>165.10 | -30 | -29 | -30 |
| 63 | Flutolanil | C_17_H_16_F_3_NO_2_ | 66332-96-5 | 14.788 | 324.10>262.10/ | -16 | -25 | -27 |
|  |  |  |  |  | 324.10>242.00 | -16 | -20 | -25 |
| 64 | Flutriafol | C_16_H_13_F_2_N_3_O | 76674-21-0 | 9.722 | 302.10>123.00/ | -15 | -28 | -22 |
|  |  |  |  |  | 302.10>109.00 | -15 | -31 | -19 |
| 65 | Fluxapyroxad | C_18_H_12_F_5_N_3_O | 907204-31-3 | 14.772 | 382.00>362.10/ | -11 | -14 | -25 |
|  |  |  |  |  | 382.00>342.10 | -11 | -21 | -22 |
| 66 | Forchlorfenuron | C_12_H_10_ClN_3_O | 68157-60-8 | 10.632 | 248.10>129.10/ | -30 | -17 | -23 |
|  |  |  |  |  | 248.10>93.10 | -30 | -34 | -17 |
| 67 | Hexaconazole | C_14_H_17_Cl_2_N_3_O | 79983-71-4 | 19.877 | 314.10>70.20/ | -15 | -21 | -28 |
|  |  |  |  |  | 314.10>159.15 | -15 | -29 | -30 |
| 68 | Hexaflumuron | C_16_H_8_Cl_2_F_6_N_2_O_3_ | 86479-06-3 | 22.107 | 459.00>438.90/ | 16 | 12 | 29 |
|  |  |  |  |  | 459.00>175.10 | 16 | 36 | 29 |
| 69 | Hexythiazox | C_17_H_21_ClN_2_O_2_S | 78587-05-0 | 23.026 | 353.10>228.00/ | -18 | -20 | -24 |
|  |  |  |  |  | 353.10>168.05 | -18 | -30 | -30 |
| 70 | Imazalil | C_14_H_14_Cl_2_N_2_O | 35554-44-0 | 11.382 | 297.00>159.00/ | -15 | -24 | -15 |
|  |  |  |  |  | 297.00>201.00 | -15 | -18 | -21 |
| 71 | Imidacloprid | C_9_H_10_ClN_5_O_2_ | 138261-41-3 | 4.684 | 256.05>175.10/ | -29 | -17 | -18 |
|  |  |  |  |  | 256.05>209.05 | -29 | -14 | -22 |
| 72 | Imidaclothiz | C_7_H_8_ClN_5_O_2_S | 105843-36-5 | 4.898 | 262.10>181.11/ | -13 | -25 | -14 |
|  |  |  |  |  | 262.10>122.00 | -10 | -40 | -15 |
| 73 | Indoxacarb | C_22_H_17_ClF_3_N_3_O_7_ | 144171-61-9 | 21.794 | 528.10>293.00/ | -26 | -15 | -21 |
|  |  |  |  |  | 528.10>249.10 | -26 | -17 | -27 |
| 74 | Ipconazole | C_18_H_24_ClN_3_O | 125225-28-7 | 21.614 | 334.20>70.10/ | -22 | -26 | -21 |
|  |  |  |  |  | 334.20>125.10 | -13 | -43 | -13 |
| 75 | Isoprothiolane | C_12_H_18_O_4_S_2_ | 50512-35-1 | 15.184 | 291.10>231.10/ | -14 | -20 | -25 |
|  |  |  |  |  | 291.10>189.10 | -14 | -30 | -20 |
| 76 | Isopyrazam | C_20_H_23_F_2_N_3_O | 881685-58-1 | 21.355 | 360.10>244.00/ | -11 | -24 | -25 |
|  |  |  |  |  | 360.10>320.10 | -11 | -21 | -22 |
| 77 | Ivermectin | C_48_H_74_O_14_ | 70288-86-7 | 24.780 | 892.50>569.20/ | -26 | -16 | -40 |
|  |  |  |  |  | 892.50>307.10 | -26 | -28 | -20 |
| 78 | Kresoxim-methyl | C_18_H_19_NO_4_ | 143390-89-0 | 19.469 | 314.10>222.20/ | -16 | -13 | -24 |
|  |  |  |  |  | 314.10>235.10 | -16 | -15 | -25 |
| 79 | Lufenuron | C_17_H_8_Cl_2_F_8_N_2_O_3_ | 103055-07-8 | 22.939 | 509.00>326.00/ | 36 | 17 | 21 |
|  |  |  |  |  | 509.00>339.00 | 36 | 11 | 22 |
| 80 | Mandipropamid | C_23_H_22_ClNO_4_ | 374726-62-2 | 15.111 | 412.10>328.05/ | -11 | -10 | -22 |
|  |  |  |  |  | 412.10>124.95 | -11 | -25 | -20 |
| 81 | Mepronil | C_17_H_19_NO_2_ | 55814-41-0 | 14.959 | 270.15>119.05/ | -30 | -25 | -30 |
|  |  |  |  |  | 270.15>228.05 | -30 | -18 | -30 |
| 82 | Metaflumizone | C_24_H_16_F_6_N_4_O_2_ | 139968-49-3 | 22.594 | 505.10>302.00/ | 24 | 21 | 30 |
|  |  |  |  |  | 505.10>285.00 | 24 | 48 | 28 |
| 83 | Metalaxyl | C_15_H_21_NO_4_ | 57837-19-1 | 10.109 | 280.10>220.20/ | -30 | -10 | -24 |
|  |  |  |  |  | 280.10>192.20 | -30 | -25 | -20 |
| 84 | Metconazole | C_17_H_22_ClN_3_O | 125116-23-6 | 20.141 | 320.00>70.10/ | -23 | -22 | -15 |
|  |  |  |  |  | 320.00>125.05 | -11 | -40 | -25 |
| 85 | Methoprene | C_19_H_34_O_3_ | 40596-69-8 | 24.148 | 279.20>191.20/ | -10 | -9 | -15 |
|  |  |  |  |  | 279.20>237.15 | -10 | -9 | -28 |
| 86 | Methoxyfenozide | C_22_H_28_N_2_O_3_ | 161050-58-4 | 15.882 | 369.20>149.10/ | -18 | -16 | -16 |
|  |  |  |  |  | 369.20>313.10 | -18 | -8 | -22 |
| 87 | Metrafenone | C_19_H_21_BrO_5_ | 220899-03-6 | 21.301 | 409.00>209.10/ | -15 | -17 | -16 |
|  |  |  |  |  | 409.00>227.10 | -20 | -22 | -18 |
| 88 | Myclobutanil | C_15_H_17_ClN_4_ | 88671-89-0 | 14.745 | 289.10>70.05/ | -30 | -21 | -28 |
|  |  |  |  |  | 289.10>125.05 | -30 | -30 | -22 |
| 89 | Novaluron | C_17_H_9_ClF_8_N_2_O_4_ | 116714-46-6 | 22.165 | 493.00>158.00/ | -15 | -18 | -28 |
|  |  |  |  |  | 493.00>141.10 | -14 | -55 | -28 |
| 90 | Oxadixyl | C_14_H_18_N_2_O_4_ | 77732-09-3 | 6.347 | 279.10>219.05/ | -30 | -18 | -23 |
|  |  |  |  |  | 279.10>133.10 | -30 | -30 | -24 |
| 91 | Paclobutrazol | C_15_H_20_ClN_3_O | 76738-62-0 | 13.974 | 294.10>70.05/ | -15 | -21 | -28 |
|  |  |  |  |  | 294.10>125.05 | -15 | -40 | -22 |
| 92 | Penconazole | C_13_H_15_Cl_2_N_3_ | 66246-88-6 | 18.484 | 284.10>70.00/ | -14 | -17 | -27 |
|  |  |  |  |  | 284.10>159.00 | -14 | -27 | -30 |
| 93 | Pencycuron | C_19_H_21_ClN_2_O | 66063-05-6 | 21.262 | 329.10>125.05/ | -17 | -15 | -22 |
|  |  |  |  |  | 329.10>218.10 | -17 | -15 | -23 |
| 94 | Penflufen | C_18_H_24_FN_3_O | 494793-67-8 | 18.655 | 318.00>141.00/ | -12 | -20 | -16 |
|  |  |  |  |  | 318.00>234.00 | -12 | -28 | -18 |
| 95 | Penthiopyrad | C_16_H_20_F_3_N_3_OS | 183675-82-3 | 19.297 | 360.00>276.00/ | -18 | -11 | -28 |
|  |  |  |  |  | 360.00>117.00 | -27 | -34 | -17 |
| 96 | Phenamacril | C_12_H_12_N_2_O_2_ | 39491-78-6 | 7.122 | 217.10>104.00/ | -11 | -40 | -20 |
|  |  |  |  |  | 217.10>189.10 | -11 | -17 | -22 |
| 97 | Phoxim | C_12_H_15_N_2_O_3_PS | 14816-18-3 | 21.168 | 299.00>77.10/ | -30 | -20 | -30 |
|  |  |  |  |  | 299.00>129.10 | -30 | -25 | -13 |
| 98 | Picoxystrobin | C_18_H_16_F_3_NO_4_ | 117428-22-5 | 19.441 | 368.10>205.10/ | -10 | -13 | -16 |
|  |  |  |  |  | 368.10>145.00 | -18 | -35 | -29 |
| 99 | Piperonylbutoxide | C_19_H_30_O_5_ | 51-03-6 | 22.717 | 356.30>177.10/ | -24 | -31 | -19 |
|  |  |  |  |  | 356.30>119.00 | -24 | -22 | -22 |
| 100 | Prochloraz | C_15_H_16_Cl_3_N_3_O_2_ | 67747-09-5 | 20.485 | 376.00>308.00/ | -19 | -11 | -21 |
|  |  |  |  |  | 376.00>266.00 | -19 | -17 | -29 |
| 101 | Prochloraz  metabolitebts 44595 | C_12_H_15_Cl_3_N_2_O_2_ | 139520-94-8 | 19.751 | 325.00>282.05/ | -11 | -15 | -21 |
|  |  |  |  |  | 325.00>284.06 | -11 | -15 | -21 |
| 102 | Prochloraz  metabolitebts 44596 | C_13_H_15_Cl_3_N_2_O_3_ | 139542-32-8 | 19.543 | 353.00>308.00/ | -12 | -14 | -17 |
|  |  |  |  |  | 353.00>310.00 | -12 | -14 | -17 |
| 103 | Propiconazole | C_15_H_17_Cl_2_N_3_O_2_ | 60207-90-1 | 19.521 | 342.05>159.10/ | -12 | -25 | -19 |
|  |  |  |  |  | 342.05>161.00 | -12 | -31 | -19 |
| 104 | Pyraclostrobin | C_19_H_18_ClN_3_O_4_ | 175013-18-0 | 21.123 | 388.10>194.05/ | -19 | -20 | -21 |
|  |  |  |  |  | 388.10>163.05 | -19 | -35 | -30 |
| 105 | Pyrametostrobin | C_21_H_23_N_3_O_4_ | 915410-70-7 | 21.184 | 382.10>194.10/ | -14 | -18 | -15 |
|  |  |  |  |  | 382.10>163.00 | -14 | -15 | -19 |
| 106 | Pyraoxystrobin | C_22_H_21_ClN_2_O_4_ | 862588-11-2 | 21.328 | 413.10>205.10/ | -15 | -18 | -16 |
|  |  |  |  |  | 413.10>145.00 | -15 | -10 | -17 |
| 107 | Pyrethrini | C_21_H_28_O_3_ | 121-21-1 | 23.399 | 329.20>161.10/ | -11 | -10 | -19 |
|  |  |  |  |  | 329.20>133.00 | -11 | -19 | -25 |
| 108 | Pyrethrinii | C_22_H_28_O_5_ | 121-29-9 | 21.916 | 373.20>161.05/ | -11 | -11 | -19 |
|  |  |  |  |  | 373.20>133.10 | -13 | -20 | -28 |
| 109 | Pyridaben | C_19_H_25_ClN_2_OS | 96489-71-3 | 23.887 | 365.10>147.10/ | -18 | -42 | -27 |
|  |  |  |  |  | 365.10>309.00 | -18 | -23 | -22 |
| 110 | Pyrimethanil | C_12_H_13_N_3_ | 53112-28-0 | 13.247 | 200.10>107.00/ | -30 | -25 | -19 |
|  |  |  |  |  | 200.10>168.10 | -30 | -29 | -30 |
| 111 | Pyrimorph | C_22_H_25_ClN_2_O_2_ | 868390-90-3 | 20.148 | 385.20>242.10/ | -14 | -27 | -18 |
|  |  |  |  |  | 385.20>272.10 | -14 | -33 | -21 |
| 112 | Pyrisoxazole | C_16_H_17_ClN_2_O | 847749-37-5 | 14.241 | 289.10>151.10/ | -11 | -14 | -18 |
|  |  |  |  |  | 289.10>120.00 | -11 | -20 | -14 |
| 113 | Sedaxane | C_18_H_19_F_2_N_3_O | 874967-67-6 | 15.483 | 332.00>159.00/ | -13 | -38 | -28 |
|  |  |  |  |  | 332.00>292.00 | -12 | -15 | -20 |
| 114 | Silthiofam | C_13_H_21_NOSSi | 175217-20-6 | 18.206 | 268.10>252.00/ | -10 | -8 | -26 |
|  |  |  |  |  | 268.10>73.10 | -10 | -27 | -30 |
| 115 | Spinetoramj | C_42_H_69_NO_10_ | 187166-40-1 | 22.282 | 748.50>142.05/ | -40 | -33 | -14 |
|  |  |  |  |  | 748.50>98.15 | -30 | -55 | -21 |
| 116 | Spinetoraml | C_43_H_69_NO_10_ | 187166-15-0 | 22.831 | 760.70>142.20/ | -22 | -32 | -25 |
|  |  |  |  |  | 760.70>98.20 | -40 | -55 | -15 |
| 117 | N-demethyl-175-J | C_41_H_67_NO_10_ | 1382419-14-8 | 22.052 | 734.50>128.15/ | -26 | -26 | -20 |
|  |  |  |  |  | 734.50>84.20 | -30 | -55 | -13 |
| 118 | N-formyl-175-J | C_42_H_67_NO_10_ | 1382419-17-1 | 23.759 | 784.50>629.30/ | -28 | -42 | -22 |
|  |  |  |  |  | 784.50>517.20 | -24 | -47 | -26 |
| 119 | Spinosad A | C_41_H_65_NO_10_ | 131929-60-7 | 21.527 | 732.40>142.00/ | -20 | -27 | -17 |
|  |  |  |  |  | 732.40>98.10 | -20 | -55 | -21 |
| 120 | Spinosad D | C_42_H_67_NO_10_ | 131929-63-0 | 22.222 | 746.40>142.01/ | -28 | -30 | -17 |
|  |  |  |  |  | 746.40>98.00 | -22 | -55 | -12 |
| 121 | Spirodiclofen | C_21_H_24_Cl_2_O_4_ | 148477-71-8 | 23.547 | 411.10>71.20/ | -21 | -16 | -28 |
|  |  |  |  |  | 411.10>313.05 | -21 | -11 | -22 |
| 122 | Spiromesifen | C_23_H_30_O_4_ | 283594-90-1 | 23.181 | 388.00>273.10/ | -14 | -15 | -21 |
|  |  |  |  |  | 388.00>255.20 | -14 | -27 | -19 |
| 123 | Spirotetramat | C_21_H_27_NO_5_ | 203313-25-1 | 16.761 | 374.00>302.00/ | -14 | -17 | -23 |
|  |  |  |  |  | 374.00>330.00 | -14 | -15 | -25 |
| 124 | Spirotetramat-enol | C_18_H_23_NO_3_ | 203312-38-3 | 9.011 | 302.10>270.00/ | -11 | -20 | -15 |
|  |  |  |  |  | 302.10>216.00 | -15 | -27 | -17 |
| 125 | Spirotetramat-keto-hydroxy | C_18_H_23_NO_4_ | 1172134-11-0 | 10.556 | 318.00>300.00/ | -12 | -13 | -23 |
|  |  |  |  |  | 318.00>214.00 | -12 | -25 | -16 |
| 126 | Spirotetramat-mono-hydroxy | C_18_H_25_NO_3_ | 1172134-12-1 | 7.029 | 304.10>254.11/ | -11 | -18 | -29 |
|  |  |  |  |  | 304.10>211.00 | -15 | -19 | -16 |
| 127 | Sulfoxaflor | C_10_H_10_F_3_N_3_OS | 946578-00-3 | 5.100 | 278.10>174.20/ | -21 | -11 | -19 |
|  |  |  |  |  | 278.10>154.10 | -20 | -26 | -25 |
| 128 | Tebuconazole | C_16_H_22_ClN_3_O | 107534-96-3 | 18.877 | 308.10>70.10/ | -11 | -23 | -15 |
|  |  |  |  |  | 308.10>125.00 | -11 | -31 | -25 |
| 129 | Tebufenozide | C_22_H_28_N_2_O_2_ | 112410-23-8 | 18.733 | 353.20>133.10/ | -18 | -20 | -24 |
|  |  |  |  |  | 353.20>297.10 | -18 | -8 | -15 |
| 130 | Teflubenzuron | C_14_H_6_Cl_2_F_4_N_2_O_2_ | 83121-18-0 | 22.593 | 379.00>339.00/ | 13 | 11 | 22 |
|  |  |  |  |  | 379.00>359.00 | 13 | 6 | 24 |
| 131 | Tetrachlorantraniliprole | C_17_H_10_BrCl_4_N_5_O_2_ | 1104384-14-6 | 17.261 | 537.85>319.85/ | -24 | -13 | -20 |
|  |  |  |  |  | 537.85>506.80 | -30 | -16 | -34 |
| 132 | Tetraconazole | C_13_H_11_Cl_2_F_4_N_3_O | 112281-77-3 | 16.518 | 372.00>159.05/ | -27 | -31 | -29 |
|  |  |  |  |  | 372.00>70.20 | -27 | -24 | -27 |
| 133 | Thiabendazole | C_10_H_7_N_3_S | 148-79-8 | 6.528 | 202.00>175.05/ | -30 | -35 | -30 |
|  |  |  |  |  | 202.00>131.10 | -30 | -25 | -24 |
| 134 | Thiacloprid | C_10_H_9_ClN_4_S | 111988-49-9 | 5.522 | 253.00>126.05/ | -28 | -30 | -22 |
|  |  |  |  |  | 253.00>99.00 | -28 | -43 | -17 |
| 135 | Thiamethoxam | C_8_H_10_ClN_5_O_3_S | 153719-23-4 | 4.312 | 292.00>211.11/ | -30 | -20 | -22 |
|  |  |  |  |  | 292.00>181.10 | -30 | -30 | -19 |
| 136 | Thidiazuron | C_9_H_8_N_4_OS | 51707-55-2 | 7.398 | 221.20>102.00/ | -15 | -16 | -18 |
|  |  |  |  |  | 221.20>128.00 | -15 | -17 | -23 |
| 137 | Thifluzamide | C_13_H_6_Br_2_F_6_N_2_O_2_S | 130000-40-7 | 17.599 | 528.80>148.00/ | -26 | -38 | -26 |
|  |  |  |  |  | 528.80>488.90 | -34 | -24 | -21 |
| 138 | Triadimefon | C_14_H_16_ClN_3_O_2_ | 43121-43-3 | 14.680 | 294.10>69.15/ | -21 | -22 | -26 |
|  |  |  |  |  | 294.10>197.05 | -21 | -15 | -21 |
| 139 | Triadimenol | C_14_H_18_ClN_3_O_2_ | 55219-65-3 | 15.163 | 296.10>70.05/ | -10 | -21 | -15 |
|  |  |  |  |  | 296.10>99.15 | -14 | -16 | -20 |
| 140 | Tricyclazole | C_9_H_7_N_3_S | 41814-78-2 | 6.070 | 190.00>163.00/ | -21 | -21 | -30 |
|  |  |  |  |  | 190.00>136.00 | -21 | -26 | -24 |
| 141 | Trifloxystrobin | C_20_H_19_F_3_N_2_O_4_ | 141517-21-7 | 21.965 | 409.10>186.05/ | -20 | -40 | -20 |
|  |  |  |  |  | 409.10>145.00 | -20 | -20 | -26 |
| 142 | Triflumizole | C_15_H_15_ClF_3_N_3_O | 99387-89-0 | 21.689 | 346.05>73.10/ | -17 | -25 | -30 |
|  |  |  |  |  | 346.05>278.00 | -17 | -22 | -30 |
| 143 | Triflumizolemetabolite  FM-6-1Ethanimidamide | C_12_H_14_ClF_3_N_2_O | 131549-75-2 | 7.861 | 295.00>43.10/ | -10 | -23 | -18 |
|  |  |  |  |  | 295.00>73.00 | -10 | -18 | -15 |
| 144 | Triflumuron | C_15_H_10_ClF_3_N_2_O_3_ | 64628-44-0 | 20.791 | 359.10>156.00/ | -17 | -16 | -30 |
|  |  |  |  |  | 359.10>139.00 | -17 | -33 | -26 |
| 145 | Triticonazole | C_17_H_20_ClN_3_O | 131983-72-7 | 16.106 | 318.10>70.05/ | -11 | -21 | -15 |
|  |  |  |  |  | 318.10>125.05 | -11 | -26 | -25 |
| 146 | Uniconazole | C_15_H_18_ClN_3_O | 83657-22-1 | 15.279 | 292.10>70.10/ | -21 | -24 | -27 |
|  |  |  |  |  | 292.10>125.00 | -21 | -28 | -23 |
| 147 | Zoxamide | C_14_H_16_Cl_3_NO_2_ | 156052-68-5 | 19.819 | 335.80>187.00/ | -16 | -24 | -20 |
|  |  |  |  |  | 335.80>159.00 | -16 | -41 | -30 |

**Table S2-1** The method validation of the matrix effects (MEs, %), average recoveries (%) and relative standard deviations (RSDs, %) of 147 pesticides and related compounds in vegetables and fruits (*n*=5).

| Pesticides | Crowndaisy | | | | Cucumber | | | | Lettuce | | | | Tomato | | | |
| --- | --- | --- | --- | --- | --- | --- | --- | --- | --- | --- | --- | --- | --- | --- | --- | --- |
|  | Average recoveries (RSDs) | | | MEs | Average recoveries (RSDs) | | | MEs | Average recoveries (RSDs) | | | MEs | Average recoveries (RSDs) | | | MEs |
|  | 0.002 mg/kg | 0.01  mg/kg | 0.1  mg/kg |  | 0.002 mg/kg | 0.01 mg/kg | 0.1 mg/kg |  | 0.002 mg/kg | 0.01  mg/kg | 0.1  mg/kg |  | 0.002 mg/kg | 0.01 mg/kg | 0.1 mg/kg |  |
| Acetamiprid | 108(9) | 96(7) | 107(6) | -2 | 83(5) | 104(6) | 79(13) | -9 | 100(2) | 96(15) | 100(9) | -9 | 107(6) | 75(8) | 88(13) | 102 |
| Albendazole | 73(2) | 93(11) | 106(17) | -28 | 84(13) | 87(12) | 88(12) | -18 | 75(4) | 74(9) | 89(3) | -32 | 74(12) | 93(10) | 95(10) | 76 |
| Ametoctradin | 85(5) | 78(8) | 94(15) | -2 | 82(9) | 87(10) | 96(15) | -14 | 82(4) | 73(9) | 110(8) | -8 | 96(10) | 109(4) | 104(14) | 113 |
| Amisulbrom | 94(11) | 90(7) | 110(14) | -15 | 88(14) | 80(5) | 81(5) | -32 | 71(16) | 74(5) | 97(8) | -16 | 89(18) | 88(4) | 90(3) | 106 |
| Azoxystrobin | 95(5) | 91(9) | 90(14) | 12 | 108(5) | 83(2) | 84(9) | -7 | 92(4) | 107(18) | 96(9) | 5 | 105(7) | 97(14) | 92(5) | 140 |
| Benalaxyl | 90(3) | 94(6) | 105(6) | 2 | 77(7) | 80(7) | 109(2) | -7 | 87(6) | 86(11) | 112(4) | -2 | 107(8) | 108(9) | 93(12) | 129 |
| Benzovindiflupyr | 80(3) | 90(4) | 86(16) | -8 | 87(9) | 82(7) | 107(15) | -20 | 80(4) | 101(7) | 90(9) | -13 | 92(11) | 92(9) | 87(1) | 110 |
| Benzoximate | 76(6) | 95(9) | 96(2) | -2 | 79(12) | 81(12) | 86(11) | -6 | 72(6) | 72(12) | 79(5) | -10 | 91(15) | 88(13) | 102(8) | 120 |
| Bitertanol | 84(9) | 80(9) | 98(5) | 0 | 79(10) | 90(12) | 92(9) | -10 | 76(7) | 103(9) | 81(7) | -2 | 100(13) | 101(5) | 106(14) | 118 |
| Boscalid | 88(3) | 85(7) | 102(5) | -14 | 92(4) | 100(2) | 78(6) | -12 | 87(4) | 97(8) | 99(8) | -18 | 106(12) | 101(5) | 108(5) | 118 |
| Bromuconazole | 91(5) | 81(11) | 105(7) | 6 | 72(10) | 78(11) | 91(4) | -10 | 85(4) | 96(18) | 112(3) | 4 | 101(6) | 103(6) | 101(8) | 60 |
| Bupirimate | 87(3) | 78(6) | 86(11) | 1 | 79(7) | 77(15) | 84(4) | -9 | 85(5) | 94(7) | 92(3) | -12 | 101(9) | 82(9) | 95(5) | 121 |
| Buprofezin | 74(10) | 93(14) | 103(5) | -15 | 78(8) | 97(15) | 78(15) | -22 | 71(5) | 96(10) | 91(9) | -14 | 97(12) | 109(10) | 77(5) | 84 |
| Carboxin | 75(10) | 81(3) | 110(1) | -8 | 78(9) | 100(15) | 88(8) | -12 | 81(2) | 89(13) | 88(9) | -13 | 78(3) | 107(7) | 87(3) | 103 |
| Chlorantraniliprole | 99(5) | 91(14) | 109(16) | -5 | 92(3) | 78(7) | 88(11) | -19 | 95(4) | 107(13) | 105(6) | -6 | 111(8) | 109(7) | 85(12) | 115 |
| Chlorfluazuron | 77(12) | 89(14) | 88(7) | -24 | 77(9) | 98(14) | 109(15) | -21 | 73(9) | 72(9) | 99(12) | -30 | 76(5) | 84(11) | 108(12) | 73 |
| Chromafenozide | 116(3) | 85(4) | 82(16) | -9 | 75(8) | 99(15) | 80(12) | -18 | 92(5) | 83(5) | 104(12) | -11 | 98(7) | 107(6) | 106(2) | 103 |
| Clothianidin | 112(9) | 78(11) | 97(10) | 3 | 88(4) | 83(9) | 81(12) | -9 | 99(3) | 106(17) | 79(11) | 0 | 110(9) | 109(12) | 78(1) | 109 |
| Coumoxystrobin | 75(8) | 90(7) | 103(3) | -21 | 78(15) | 110(4) | 85(11) | -27 | 79(4) | 92(16) | 81(7) | -31 | 72(11) | 108(8) | 89(11) | 91 |
| Cyantraniliprole | 98(5) | 85(3) | 104(9) | 5 | 89(4) | 108(15) | 90(1) | -16 | 93(5) | 96(6) | 78(3) | -9 | 102(12) | 107(4) | 108(13) | 115 |
| Cyazofamid | 84(3) | 88(5) | 108(14) | -11 | 75(9) | 103(13) | 85(3) | -28 | 79(4) | 80(12) | 99(9) | -31 | 106(12) | 88(6) | 104(5) | 64 |
| CCIM (4-Chlor-2-cyano-5-  (4-methylphenyl)imidazol) | 91(5) | 84(9) | 105(1) | 19 | 74(6) | 81(1) | 102(4) | -1 | 88(5) | 93(5) | 85(5) | 14 | 105(7) | 91(14) | 99(13) | 146 |
| Cyetpyrafen | 75(3) | 94(13) | 97(11) | -41 | 82(13) | 80(6) | 76(12) | -35 | 72(6) | 72(9) | 79(10) | -42 | 86(9) | 79(11) | 95(11) | 84 |
| Cyflufenamid | 78(5) | 81(6) | 101(10) | -6 | 85(10) | 92(6) | 84(11) | -19 | 77(4) | 89(17) | 87(4) | -7 | 89(11) | 76(3) | 93(13) | 89 |
| Cyflumetofen | 73(7) | 82(5) | 105(17) | -81 | 73(10) | 87(12) | 102(8) | -38 | 75(12) | 75(12) | 79(6) | -48 | 86(8) | 89(9) | 85(3) | 71 |
| Cymoxanil | 104(4) | 93(10) | 79(6) | -70 | 85(4) | 81(2) | 94(13) | -10 | 96(4) | 74(16) | 85(7) | -32 | 103(8) | 78(1) | 79(7) | 106 |
| Cyproconazole | 97(3) | 94(5) | 101(16) | 12 | 85(6) | 97(3) | 90(2) | -5 | 90(5) | 78(14) | 105(12) | 7 | 110(9) | 76(3) | 106(2) | 119 |
| Cyprodinil | 75(3) | 79(7) | 90(1) | 8 | 86(9) | 103(3) | 76(4) | -3 | 73(3) | 96(8) | 105(12) | 6 | 84(2) | 88(10) | 91(8) | 94 |
| Diclobutrazol | 94(3) | 94(11) | 96(4) | 4 | 75(5) | 107(14) | 90(10) | -8 | 86(4) | 88(6) | 84(12) | 1 | 102(10) | 99(5) | 92(14) | 124 |
| Diethofencarb | 98(4) | 88(5) | 81(4) | 5 | 86(5) | 88(9) | 76(9) | -7 | 92(3) | 105(15) | 83(12) | 2 | 113(11) | 81(3) | 90(4) | 124 |
| Difenoconazole | 79(7) | 88(6) | 77(2) | -1 | 75(8) | 98(6) | 108(2) | -14 | 78(3) | 94(15) | 81(7) | -3 | 84(4) | 81(4) | 81(11) | 104 |
| Diflubenzuron | 78(3) | 86(3) | 109(14) | -11 | 77(6) | 83(13) | 80(3) | -2 | 78(4) | 75(9) | 91(9) | -9 | 87(1) | 102(8) | 83(14) | 105 |
| Dimethomorph | 104(10) | 87(5) | 87(2) | -2 | 90(5) | 84(6) | 87(9) | -19 | 99(5) | 72(7) | 79(9) | -12 | 114(10) | 91(4) | 89(14) | 137 |
| Dimoxystrobin | 95(1) | 79(9) | 92(11) | -8 | 80(6) | 90(9) | 77(3) | -12 | 89(5) | 74(12) | 98(10) | -21 | 108(9) | 83(8) | 95(4) | 108 |
| Diniconazole | 83(4) | 80(11) | 82(5) | 2 | 73(11) | 77(13) | 78(3) | -6 | 83(6) | 74(11) | 97(6) | 6 | 104(11) | 78(6) | 105(11) | 120 |
| Emamectinbenzoate | 95(8) | 94(7) | 103(10) | -42 | 79(10) | 94(7) | 95(9) | -21 | 98(6) | 95(7) | 102(10) | -54 | 104(12) | 85(15) | 91(12) | 130 |
| Enestroburin | 77(7) | 93(12) | 79(13) | -4 | 76(11) | 96(3) | 105(3) | -18 | 78(12) | 105(12) | 79(12) | -7 | 85(6) | 105(3) | 75(1) | 104 |
| Epoxiconazole | 78(8) | 91(13) | 84(4) | 5 | 74(4) | 106(7) | 78(12) | -8 | 84(4) | 98(10) | 88(7) | -1 | 104(10) | 86(1) | 78(13) | 135 |
| Ethiprole | 100(2) | 79(4) | 98(16) | -2 | 92(6) | 78(5) | 93(15) | -6 | 94(5) | 96(16) | 88(11) | 5 | 112(13) | 86(6) | 106(6) | 117 |
| Ethirimol | 91(2) | 82(5) | 110(16) | -7 | 79(6) | 78(7) | 82(11) | -14 | 90(3) | 102(8) | 103(5) | -12 | 109(10) | 80(12) | 76(5) | 114 |
| Famoxadone | 79(15) | 88(5) | 76(9) | -31 | 73(2) | 109(2) | 84(2) | -22 | 76(11) | 90(18) | 103(5) | -19 | 76(9) | 88(7) | 82(14) | 114 |
| Fenamidone | 94(2) | 88(11) | 76(12) | 4 | 81(6) | 104(14) | 96(6) | -10 | 88(4) | 74(6) | 101(12) | 0 | 105(11) | 86(5) | 79(3) | 124 |
| Fenarimol | 85(4) | 84(11) | 108(1) | -10 | 74(7) | 76(8) | 95(4) | 0 | 82(4) | 105(6) | 97(5) | 11 | 116(9) | 103(12) | 100(1) | 90 |
| Fenbuconazole | 87(4) | 82(10) | 106(13) | -9 | 78(6) | 80(9) | 77(1) | -15 | 83(4) | 89(12) | 88(7) | -1 | 99(10) | 75(8) | 95(6) | 111 |
| Fenhexamid | 92(1) | 90(8) | 93(1) | 8 | 81(5) | 93(8) | 87(4) | -13 | 88(6) | 77(15) | 91(11) | 30 | 101(11) | 79(5) | 76(12) | 118 |
| Fenoxanil | 92(2) | 79(5) | 99(9) | -8 | 76(6) | 95(9) | 86(11) | -16 | 82(5) | 84(9) | 84(9) | -11 | 102(12) | 102(14) | 104(8) | 103 |
| Fenpropidin | 94(8) | 95(4) | 107(7) | -2 | 93(12) | 106(12) | 84(3) | -11 | 91(4) | 100(14) | 108(12) | -3 | 102(12) | 99(13) | 96(3) | 141 |
| Fenpropimorph | 87(1) | 86(11) | 101(14) | -7 | 83(4) | 102(14) | 110(10) | -17 | 88(3) | 72(14) | 90(9) | -11 | 89(5) | 85(6) | 90(4) | 113 |
| Fenpyrazamine | 95(2) | 89(9) | 108(9) | -1 | 76(6) | 89(13) | 107(9) | -6 | 83(6) | 81(10) | 87(5) | -11 | 106(8) | 87(8) | 92(13) | 127 |
| Fipronil | 97(3) | 88(9) | 103(7) | 12 | 72(5) | 83(1) | 82(9) | -6 | 88(5) | 107(11) | 111(8) | 18 | 99(9) | 95(11) | 93(12) | 142 |
| Fipronil-desulfinyl | 88(4) | 85(4) | 90(14) | 12 | 76(7) | 86(14) | 98(13) | -4 | 83(5) | 87(18) | 86(12) | 8 | 95(10) | 105(12) | 76(8) | 144 |
| Fipronil-sulfide | 83(3) | 78(3) | 96(17) | 3 | 72(7) | 76(6) | 106(4) | -16 | 81(6) | 98(15) | 80(6) | -1 | 94(10) | 97(5) | 104(12) | 123 |
| Fipronil-sulfone | 83(3) | 82(11) | 88(14) | -8 | 79(9) | 79(2) | 109(13) | -20 | 82(4) | 86(10) | 104(8) | -7 | 89(10) | 79(14) | 78(10) | 119 |
| Fluazinam | 79(7) | 90(11) | 110(13) | 0 | 77(14) | 107(11) | 94(3) | -24 | 71(5) | 94(6) | 94(9) | -6 | 78(8) | 97(15) | 75(5) | 112 |
| Flubendiamide | 88(2) | 81(4) | 86(4) | 47 | 80(8) | 93(9) | 104(12) | 0 | 83(5) | 88(11) | 81(12) | 23 | 97(10) | 78(5) | 93(6) | 165 |
| Fludioxonil | 79(5) | 80(13) | 76(3) | -15 | 76(2) | 102(1) | 83(13) | -27 | 80(4) | 73(8) | 93(12) | -1 | 81(15) | 98(2) | 77(1) | 75 |
| Flufenoxuron | 75(8) | 85(9) | 104(4) | -60 | 76(6) | 102(12) | 109(12) | -64 | 73(6) | 73(7) | 92(11) | -61 | 74(6) | 89(2) | 85(14) | -10 |
| Flumetralin | 72(4) | 84(13) | 97(11) | -18 | 82(11) | 77(13) | 80(3) | -30 | 73(4) | 105(9) | 93(3) | -21 | 77(15) | 95(14) | 93(7) | 90 |
| Flumorph | 98(6) | 89(9) | 80(10) | 1 | 91(4) | 94(10) | 79(7) | -9 | 92(4) | 108(9) | 92(11) | -6 | 111(10) | 109(8) | 92(5) | 123 |
| Fluopicolide | 93(3) | 78(11) | 89(17) | -1 | 81(3) | 104(3) | 103(8) | -15 | 91(3) | 105(15) | 92(11) | -8 | 106(11) | 86(14) | 77(10) | 109 |
| Fluopyram | 96(2) | 85(7) | 102(12) | 4 | 83(6) | 87(14) | 109(10) | -7 | 105(6) | 96(5) | 87(4) | 3 | 106(10) | 87(2) | 86(4) | 134 |
| Flusilazole | 81(4) | 96(11) | 103(8) | 7 | 76(7) | 104(15) | 106(13) | -7 | 83(5) | 83(10) | 102(5) | -8 | 101(10) | 77(15) | 82(5) | 122 |
| Flutolanil | 92(2) | 85(12) | 82(15) | 9 | 78(6) | 98(15) | 102(6) | -16 | 87(5) | 106(10) | 108(5) | -12 | 103(10) | 82(1) | 81(11) | 129 |
| Flutriafol | 97(3) | 88(8) | 85(15) | 15 | 92(3) | 104(11) | 99(10) | -9 | 93(4) | 80(9) | 104(7) | 5 | 112(10) | 90(7) | 83(10) | 133 |
| Fluxapyroxad | 93(3) | 81(4) | 101(11) | -7 | 81(6) | 82(3) | 104(10) | -17 | 89(4) | 81(16) | 78(11) | -10 | 106(10) | 104(14) | 96(4) | 89 |
| Forchlorfenuron | 81(3) | 78(10) | 91(7) | 9 | 74(4) | 103(6) | 96(2) | -8 | 82(4) | 93(17) | 107(12) | 5 | 100(7) | 84(7) | 87(5) | 130 |
| Hexaconazole | 93(5) | 79(9) | 78(3) | -6 | 77(8) | 76(11) | 108(1) | -8 | 89(4) | 99(9) | 85(12) | 2 | 103(11) | 78(14) | 94(5) | 118 |
| Hexaflumuron | 75(8) | 88(12) | 87(12) | 24 | 89(8) | 101(7) | 101(6) | -12 | 77(4) | 76(10) | 95(3) | 18 | 77(9) | 110(8) | 86(10) | 135 |
| Hexythiazox | 74(7) | 93(13) | 94(10) | -6 | 84(12) | 97(6) | 77(9) | -18 | 72(3) | 94(16) | 90(10) | -17 | 75(7) | 97(8) | 102(10) | 113 |
| Imazalil | 77(10) | 84(11) | 86(2) | -4 | 79(5) | 92(6) | 83(6) | -14 | 85(4) | 72(9) | 80(7) | -9 | 105(11) | 76(15) | 101(11) | 112 |
| Imidacloprid | 76(5) | 83(7) | 83(6) | 8 | 88(3) | 110(3) | 102(10) | -4 | 112(4) | 104(6) | 83(11) | 3 | 107(7) | 104(13) | 97(5) | 116 |
| Imidaclothiz | 92(5) | 95(4) | 101(7) | 9 | 84(3) | 109(4) | 85(7) | -6 | 96(3) | 75(12) | 111(5) | 6 | 114(6) | 110(13) | 78(9) | 121 |
| Indoxacarb | 75(14) | 96(9) | 104(15) | -23 | 80(10) | 106(3) | 105(11) | -34 | 76(5) | 91(16) | 102(6) | -21 | 85(7) | 82(10) | 87(5) | 77 |
| Ipconazole | 83(4) | 92(11) | 84(4) | -2 | 74(9) | 85(5) | 90(13) | -11 | 79(3) | 89(5) | 101(10) | -5 | 94(10) | 107(12) | 100(7) | 132 |
| Isoprothiolane | 83(2) | 91(4) | 86(8) | -14 | 77(5) | 107(12) | 75(1) | -13 | 86(4) | 101(8) | 91(8) | 3 | 106(9) | 107(11) | 107(4) | 117 |
| Isopyrazam | 79(3) | 80(13) | 101(12) | -1 | 80(11) | 96(15) | 108(1) | -15 | 82(6) | 94(16) | 111(10) | -3 | 97(10) | 96(3) | 82(8) | 103 |
| Ivermectin | 87(12) | 81(7) | 105(6) | -57 | 76(19) | 99(6) | 90(3) | -52 | 82(10) | 85(16) | 81(7) | -52 | 76(19) | 93(6) | 80(8) | 43 |
| Kresoxim-methyl | 87(3) | 87(3) | 90(1) | 24 | 76(2) | 110(13) | 87(4) | 15 | 80(4) | 98(12) | 103(6) | 20 | 89(2) | 101(8) | 82(11) | 159 |
| Lufenuron | 85(4) | 91(8) | 109(6) | -7 | 82(6) | 100(9) | 95(6) | -24 | 78(12) | 102(8) | 98(12) | -1 | 81(11) | 93(3) | 101(7) | 92 |
| Mandipropamid | 94(2) | 88(8) | 105(3) | -11 | 78(5) | 75(8) | 104(2) | -16 | 82(4) | 75(8) | 85(7) | -11 | 108(11) | 104(10) | 80(10) | 105 |
| Mepronil | 90(2) | 89(8) | 85(3) | -1 | 77(5) | 76(15) | 77(2) | -10 | 85(4) | 103(18) | 110(8) | -5 | 104(11) | 90(1) | 87(5) | 118 |
| Metaflumizone | 72(5) | 81(11) | 93(2) | 1 | 76(13) | 110(15) | 82(3) | -14 | 76(5) | 72(7) | 104(3) | -6 | 74(12) | 102(4) | 79(13) | 95 |
| Metalaxyl | 99(4) | 78(11) | 109(7) | 3 | 91(3) | 84(4) | 82(11) | -10 | 95(4) | 104(9) | 110(8) | -4 | 111(10) | 105(4) | 102(4) | 122 |
| Metconazole | 90(3) | 94(9) | 92(3) | 14 | 78(7) | 100(15) | 79(3) | -3 | 84(4) | 106(11) | 81(3) | 9 | 104(8) | 89(9) | 104(14) | 136 |
| Methoprene | 83(11) | 91(13) | 80(10) | -28 | 76(12) | 76(12) | 88(10) | -32 | 88(4) | 102(8) | 92(5) | -31 | 77(17) | 109(11) | 79(1) | 70 |
| Methoxyfenozide | 95(3) | 92(10) | 105(5) | 6 | 83(4) | 109(2) | 76(8) | -26 | 89(4) | 100(18) | 94(4) | -9 | 107(10) | 94(10) | 105(8) | 81 |
| Metrafenone | 80(3) | 91(9) | 93(8) | -4 | 78(10) | 101(7) | 108(15) | -17 | 77(4) | 75(5) | 102(7) | -8 | 91(7) | 83(15) | 107(10) | 94 |
| Myclobutanil | 94(4) | 80(8) | 84(2) | 8 | 84(4) | 96(3) | 90(11) | -6 | 88(5) | 73(17) | 104(7) | -11 | 106(9) | 84(5) | 108(2) | 128 |
| Novaluron | 79(9) | 79(11) | 96(12) | -20 | 74(8) | 108(10) | 100(8) | -32 | 79(5) | 93(7) | 102(5) | -23 | 81(8) | 94(1) | 102(12) | 83 |
| Oxadixyl | 101(6) | 86(8) | 76(16) | -16 | 93(3) | 110(3) | 99(9) | -18 | 99(5) | 72(12) | 99(7) | -8 | 107(10) | 104(4) | 84(14) | 71 |
| Paclobutrazol | 95(2) | 79(12) | 77(5) | 5 | 89(6) | 109(13) | 78(2) | -10 | 90(4) | 100(11) | 108(11) | 2 | 110(12) | 81(9) | 85(7) | 116 |
| Penconazole | 88(2) | 96(11) | 88(15) | -14 | 79(10) | 110(15) | 106(4) | -27 | 82(6) | 81(14) | 95(11) | -24 | 97(7) | 107(13) | 101(14) | 83 |
| Pencycuron | 79(4) | 82(11) | 101(11) | -2 | 77(10) | 100(15) | 98(4) | -14 | 75(5) | 108(12) | 97(7) | -6 | 89(9) | 108(7) | 90(4) | 100 |
| Penflufen | 90(3) | 79(5) | 111(14) | 1 | 80(7) | 78(14) | 110(3) | -10 | 86(4) | 102(16) | 102(11) | -4 | 111(11) | 93(9) | 84(6) | 128 |
| Penthiopyrad | 93(3) | 81(11) | 83(9) | -2 | 78(8) | 86(12) | 108(11) | -11 | 85(5) | 102(11) | 105(11) | -7 | 104(9) | 85(14) | 99(8) | 129 |
| Phenamacril | 91(3) | 79(3) | 85(1) | 1 | 88(3) | 86(5) | 86(3) | -11 | 93(3) | 73(7) | 107(6) | -5 | 103(15) | 102(14) | 99(1) | 112 |
| Phoxim | 71(7) | 78(3) | 80(12) | -11 | 80(10) | 110(10) | 84(4) | -20 | 79(4) | 97(7) | 80(3) | -8 | 77(6) | 100(5) | 88(12) | 83 |
| Picoxystrobin | 86(2) | 84(8) | 101(2) | -9 | 78(10) | 89(1) | 108(7) | -22 | 88(5) | 79(16) | 77(9) | -22 | 101(10) | 104(10) | 103(4) | 105 |
| Piperonylbutoxide | 78(6) | 87(13) | 77(12) | -16 | 74(7) | 77(12) | 79(3) | -24 | 74(2) | 83(9) | 93(11) | -14 | 80(5) | 106(4) | 102(3) | 90 |
| Prochloraz | 79(3) | 86(6) | 78(2) | 0 | 80(8) | 78(8) | 102(11) | -14 | 78(4) | 103(11) | 106(3) | -7 | 96(7) | 109(13) | 95(8) | 118 |
| Prochlorazmetabolitebts44595 | 85(3) | 85(5) | 80(10) | 5 | 71(8) | 99(14) | 90(10) | -10 | 85(4) | 91(12) | 111(6) | -9 | 102(10) | 90(1) | 93(6) | 111 |
| Prochlorazmetabolitebts44596 | 88(3) | 87(10) | 89(6) | 26 | 73(13) | 79(1) | 101(12) | 3 | 81(4) | 80(13) | 86(8) | 6 | 105(9) | 81(14) | 77(3) | 175 |
| Propiconazole | 111(17) | 88(9) | 107(7) | -2 | 84(5) | 101(9) | 78(2) | -3 | 104(6) | 95(12) | 89(7) | 6 | 104(15) | 104(6) | 95(4) | 103 |
| Pyraclostrobin | 78(11) | 94(6) | 100(16) | 1 | 73(6) | 93(9) | 88(8) | -9 | 71(3) | 89(16) | 112(8) | -8 | 80(8) | 106(8) | 98(4) | 120 |
| Pyrametostrobin | 79(2) | 94(5) | 105(11) | -4 | 74(9) | 89(12) | 88(12) | -15 | 82(4) | 101(9) | 77(9) | -7 | 90(6) | 91(14) | 98(4) | 104 |
| Pyraoxystrobin | 76(6) | 94(12) | 82(9) | 0 | 73(1) | 79(1) | 103(1) | -16 | 71(3) | 104(12) | 104(4) | -7 | 72(4) | 80(3) | 99(2) | 115 |
| Pyrethrini | 75(5) | 92(14) | 86(8) | -15 | 74(6) | 110(3) | 81(8) | -28 | 74(2) | 85(13) | 102(8) | -23 | 75(13) | 110(12) | 87(11) | 97 |
| Pyrethrinii | 77(12) | 83(14) | 97(9) | -5 | 71(1) | 88(15) | 108(2) | -21 | 74(8) | 105(12) | 79(3) | -13 | 86(7) | 76(1) | 96(12) | 115 |
| Pyridaben | 103(4) | 83(6) | 89(4) | -22 | 72(2) | 83(7) | 93(8) | -27 | 75(5) | 100(17) | 89(6) | -28 | 75(4) | 101(4) | 97(14) | 85 |
| Pyrimethanil | 98(5) | 78(13) | 91(5) | -2 | 74(4) | 87(14) | 90(14) | -26 | 88(3) | 93(14) | 104(3) | -7 | 108(5) | 102(12) | 79(14) | 88 |
| Pyrimorph | 89(3) | 96(8) | 84(2) | 0 | 73(7) | 83(7) | 86(5) | -13 | 86(5) | 75(5) | 86(8) | -6 | 102(9) | 106(14) | 98(11) | 119 |
| Pyrisoxazole | 86(4) | 94(5) | 81(7) | -9 | 83(7) | 100(13) | 89(3) | -9 | 88(5) | 90(12) | 78(9) | -13 | 103(10) | 109(13) | 107(12) | 128 |
| Sedaxane | 89(2) | 94(14) | 99(5) | 0 | 78(7) | 90(8) | 76(4) | -10 | 87(5) | 100(11) | 100(12) | -5 | 105(10) | 75(10) | 81(11) | 131 |
| Silthiofam | 96(2) | 82(3) | 76(13) | -1 | 77(5) | 79(13) | 79(15) | -7 | 88(4) | 95(16) | 107(6) | -10 | 107(10) | 85(1) | 92(9) | 124 |
| Spinetoramj | 87(7) | 96(11) | 76(8) | -33 | 91(8) | 75(9) | 97(10) | -32 | 94(12) | 76(5) | 85(10) | -50 | 99(8) | 75(2) | 94(8) | 91 |
| Spinetoraml | 78(3) | 92(11) | 77(14) | -64 | 73(6) | 95(1) | 86(8) | -33 | 75(9) | 100(18) | 85(5) | -72 | 104(8) | 100(12) | 81(12) | 85 |
| N-demethyl-175-J | 90(3) | 93(11) | 100(17) | 37 | 83(8) | 87(10) | 109(8) | 41 | 96(6) | 73(11) | 102(8) | 14 | 114(8) | 104(1) | 76(10) | 317 |
| N-formyl-175-J | 87(4) | 95(13) | 97(14) | 4 | 72(12) | 77(14) | 104(14) | -20 | 80(5) | 77(9) | 111(4) | -8 | 94(13) | 81(6) | 82(6) | 111 |
| Spinosad A | 96(5) | 87(13) | 94(8) | -31 | 78(16) | 88(11) | 106(6) | -23 | 87(8) | 93(14) | 77(9) | -48 | 88(15) | 108(4) | 87(12) | 105 |
| Spinosad D | 83(5) | 78(11) | 95(17) | -30 | 71(5) | 79(15) | 84(10) | -26 | 81(4) | 78(13) | 103(6) | -48 | 95(6) | 98(12) | 104(2) | 114 |
| Spirodiclofen | 107(10) | 78(4) | 89(11) | -23 | 79(15) | 110(6) | 75(10) | -26 | 78(8) | 93(12) | 81(11) | -38 | 75(8) | 103(15) | 91(11) | 95 |
| Spiromesifen | 77(11) | 91(12) | 110(5) | 0 | 78(8) | 102(3) | 90(8) | -27 | 77(15) | 97(8) | 101(10) | -19 | 104(15) | 77(13) | 93(7) | 100 |
| Spirotetramat | 94(4) | 90(7) | 101(7) | -3 | 89(4) | 84(6) | 107(10) | 2 | 89(5) | 78(10) | 104(10) | -4 | 118(10) | 78(4) | 100(10) | 160 |
| Spirotetramat-enol | 99(6) | 90(3) | 88(8) | 69 | 88(4) | 99(4) | 108(15) | 2 | 100(5) | 107(16) | 105(10) | 48 | 113(7) | 89(10) | 106(9) | 133 |
| Spirotetramat-keto-hydroxy | 105(6) | 81(14) | 105(8) | 8 | 102(3) | 108(8) | 82(6) | -8 | 96(4) | 82(15) | 84(7) | -2 | 116(11) | 91(1) | 85(12) | 139 |
| Spirotetramat-mono-hydroxy | 104(5) | 79(5) | 92(15) | 3 | 100(4) | 86(2) | 96(14) | -13 | 98(5) | 83(14) | 108(3) | -3 | 118(11) | 110(4) | 75(11) | 122 |
| Sulfoxaflor | 98(3) | 79(12) | 105(4) | -6 | 82(5) | 101(4) | 81(14) | -3 | 96(3) | 93(9) | 103(7) | -16 | 108(10) | 97(15) | 83(3) | 113 |
| Tebuconazole | 90(3) | 96(3) | 102(3) | 2 | 75(7) | 110(2) | 82(9) | -25 | 107(7) | 94(7) | 105(5) | 0 | 103(9) | 79(3) | 87(5) | 84 |
| Tebufenozide | 92(1) | 92(5) | 83(4) | 3 | 77(6) | 78(11) | 77(2) | -13 | 85(4) | 81(8) | 84(7) | -8 | 110(12) | 97(8) | 84(7) | 118 |
| Teflubenzuron | 75(10) | 82(9) | 79(7) | 39 | 76(7) | 97(4) | 79(14) | 0 | 72(5) | 104(17) | 86(11) | 30 | 75(16) | 101(13) | 101(12) | 112 |
| Tetrachlorantraniliprole | 110(3) | 78(8) | 99(17) | -10 | 74(11) | 105(8) | 76(6) | -15 | 87(6) | 73(10) | 77(11) | -35 | 97(10) | 89(4) | 95(3) | 47 |
| Tetraconazole | 88(2) | 78(11) | 94(1) | 6 | 72(6) | 96(10) | 90(10) | -12 | 87(5) | 87(10) | 112(3) | -6 | 81(6) | 77(2) | 80(4) | 125 |
| Thiabendazole | 75(8) | 84(14) | 85(2) | -30 | 80(4) | 78(11) | 101(1) | -22 | 96(4) | 94(8) | 77(5) | -11 | 107(7) | 94(7) | 80(3) | 105 |
| Thiacloprid | 92(3) | 93(4) | 109(10) | -2 | 82(4) | 76(7) | 75(4) | -12 | 92(3) | 89(16) | 94(8) | -6 | 108(8) | 95(8) | 94(7) | 105 |
| Thiamethoxam | 103(11) | 95(8) | 91(15) | -5 | 93(3) | 107(3) | 91(2) | -15 | 93(4) | 81(10) | 107(5) | -15 | 115(8) | 109(15) | 88(2) | 76 |
| Thidiazuron | 84(5) | 87(8) | 100(7) | 26 | 74(4) | 102(13) | 89(3) | -5 | 86(4) | 90(7) | 112(10) | 9 | 107(11) | 78(9) | 75(3) | 111 |
| Thifluzamide | 94(5) | 84(4) | 106(4) | 0 | 72(5) | 94(8) | 82(8) | -8 | 86(5) | 78(9) | 80(3) | -7 | 84(7) | 76(11) | 97(6) | 101 |
| Triadimefon | 96(3) | 79(6) | 110(5) | 10 | 89(5) | 109(8) | 90(4) | -8 | 87(5) | 79(6) | 79(9) | 0 | 106(10) | 100(3) | 90(10) | 123 |
| Triadimenol | 89(6) | 91(5) | 90(1) | 6 | 93(13) | 105(4) | 85(2) | -17 | 88(4) | 77(12) | 77(5) | -2 | 109(13) | 83(8) | 86(13) | 124 |
| Tricyclazole | 89(2) | 92(10) | 97(5) | -7 | 81(6) | 87(9) | 95(7) | -12 | 89(4) | 97(8) | 79(3) | -8 | 110(8) | 97(4) | 76(6) | 110 |
| Trifloxystrobin | 79(7) | 88(3) | 100(2) | 0 | 77(13) | 100(12) | 81(10) | -14 | 75(4) | 86(15) | 100(7) | -5 | 90(7) | 82(10) | 105(2) | 120 |
| Triflumizole | 75(13) | 83(14) | 105(17) | 4 | 79(7) | 107(2) | 77(13) | -10 | 79(7) | 108(10) | 111(11) | -1 | 98(15) | 98(15) | 89(6) | 116 |
| Triflumizolemetabolite  FM-6-1Ethanimidamide | 97(4) | 82(5) | 78(11) | -5 | 88(4) | 89(6) | 103(15) | -17 | 94(3) | 92(8) | 102(6) | -16 | 108(9) | 99(10) | 80(8) | 110 |
| Triflumuron | 77(6) | 82(13) | 101(4) | 0 | 72(10) | 87(14) | 87(7) | -13 | 77(4) | 87(6) | 105(10) | -9 | 80(8) | 102(5) | 91(14) | 91 |
| Triticonazole | 91(4) | 84(7) | 85(16) | 0 | 83(5) | 98(11) | 97(7) | 7 | 89(5) | 73(15) | 78(5) | -3 | 113(11) | 88(7) | 90(14) | 109 |
| Uniconazole | 92(3) | 92(7) | 77(7) | 12 | 84(6) | 94(12) | 79(9) | -6 | 90(6) | 77(9) | 92(5) | 7 | 110(8) | 75(8) | 90(3) | 119 |
| Zoxamide | 85(3) | 86(7) | 110(7) | 2 | 76(9) | 91(7) | 110(10) | -8 | 83(5) | 80(14) | 106(12) | -4 | 96(9) | 107(9) | 81(13) | 115 |

**Table S2-2** The method validation of the matrix effects (MEs, %), average recoveries (%) and relative standard deviations (RSDs, %) of 147 pesticides and related compounds in vegetables and fruits (*n*=5).

| Pesticides | Blueberry | | | | Peach | | | | Pear | | | | Strawberry | | | | Grape | | | |
| --- | --- | --- | --- | --- | --- | --- | --- | --- | --- | --- | --- | --- | --- | --- | --- | --- | --- | --- | --- | --- |
|  | Average recoveries (RSDs) | | | MEs | Average recoveries (RSDs) | | | MEs | Average recoveries (RSDs) | | | MEs | Average recoveries (RSDs) | | | MEs | Average recoveries (RSDs) | | | MEs |
|  | 0.002 mg/kg | 0.01 mg/kg | 0.1 mg/kg |  | 0.002 mg/kg | 0.01 mg/kg | 0.1 mg/kg |  | 0.002 mg/kg | 0.01 mg/kg | 0.1 mg/kg |  | 0.002 mg/kg | 0.01 mg/kg | 0.1 mg/kg |  | 0.002 mg/kg | 0.01 mg/kg | 0.1 mg/kg |  |
| Acetamiprid | 105(7) | 78(3) | 112(8) | 6 | 92(3) | 99(9) | 104(17) | -4 | 97(6) | 80(16) | 84(7) | 3 | 88(4) | 77(8) | 78(11) | 8 | 97(5) | 74(13) | 105(10) | 11 |
| Albendazole | 87(7) | 71(3) | 74(10) | -21 | 76(8) | 103(7) | 99(10) | -27 | 76(8) | 82(14) | 84(5) | -7 | 78(9) | 80(3) | 98(3) | -20 | 73(5) | 109(13) | 94(14) | -7 |
| Ametoctradin | 88(6) | 90(13) | 100(9) | 2 | 93(5) | 96(1) | 112(4) | -12 | 91(7) | 75(6) | 92(12) | -5 | 83(3) | 104(3) | 86(9) | 5 | 91(3) | 75(15) | 79(7) | 14 |
| Amisulbrom | 83(5) | 75(6) | 77(9) | -10 | 81(9) | 78(3) | 110(14) | -33 | 97(8) | 101(9) | 93(7) | -30 | 73(11) | 97(2) | 96(9) | -2 | 72(6) | 93(7) | 105(3) | 10 |
| Azoxystrobin | 92(2) | 102(4) | 88(12) | 18 | 98(5) | 89(9) | 82(16) | -3 | 94(7) | 101(16) | 76(7) | 2 | 86(9) | 97(7) | 85(8) | 14 | 92(8) | 100(16) | 84(4) | 23 |
| Benalaxyl | 94(6) | 103(15) | 87(6) | 8 | 93(8) | 93(1) | 96(17) | -7 | 95(7) | 84(5) | 83(4) | -1 | 84(5) | 109(6) | 85(13) | 11 | 91(4) | 90(13) | 84(14) | 20 |
| Benzovindiflupyr | 90(6) | 78(14) | 80(3) | 0 | 88(8) | 87(10) | 91(15) | -16 | 90(5) | 93(8) | 72(4) | -12 | 84(4) | 98(7) | 110(13) | -2 | 84(6) | 73(4) | 92(8) | 11 |
| Benzoximate | 82(6) | 81(6) | 86(5) | 24 | 81(8) | 103(10) | 107(1) | -10 | 84(8) | 99(12) | 79(9) | -7 | 75(8) | 82(6) | 79(6) | 13 | 81(10) | 95(17) | 102(8) | 21 |
| Bitertanol | 96(6) | 101(9) | 97(4) | 10 | 91(7) | 95(5) | 103(9) | -9 | 104(8) | 72(10) | 89(5) | 0 | 90(6) | 77(10) | 78(3) | 9 | 96(8) | 108(5) | 85(11) | 17 |
| Boscalid | 93(6) | 97(12) | 88(7) | 3 | 91(6) | 86(8) | 78(16) | -9 | 92(7) | 84(15) | 81(7) | -5 | 100(9) | 92(10) | 73(7) | -10 | 91(12) | 86(9) | 78(7) | 17 |
| Bromuconazole | 89(8) | 102(10) | 98(5) | 14 | 95(4) | 97(8) | 88(2) | -4 | 93(7) | 72(2) | 93(16) | -23 | 89(6) | 105(1) | 85(5) | 10 | 86(4) | 89(17) | 75(8) | 18 |
| Bupirimate | 93(6) | 88(9) | 88(13) | 9 | 89(6) | 105(3) | 86(11) | -10 | 93(6) | 86(14) | 91(13) | -1 | 88(3) | 86(1) | 91(13) | 4 | 88(5) | 108(16) | 93(6) | 20 |
| Buprofezin | 82(6) | 81(2) | 111(4) | -8 | 84(9) | 92(1) | 111(2) | -20 | 89(5) | 104(16) | 79(16) | -16 | 90(9) | 87(7) | 94(9) | -8 | 89(6) | 109(16) | 87(6) | 1 |
| Carboxin | 90(7) | 96(15) | 76(7) | -5 | 72(2) | 104(5) | 79(16) | -8 | 79(7) | 92(6) | 85(16) | -4 | 77(10) | 98(4) | 110(13) | -1 | 78(4) | 101(9) | 110(3) | 9 |
| Chlorantraniliprole | 97(6) | 75(5) | 105(11) | -1 | 91(6) | 103(2) | 79(8) | -18 | 98(5) | 81(9) | 90(14) | -9 | 89(5) | 89(7) | 104(7) | 2 | 91(3) | 105(2) | 77(10) | 14 |
| Chlorfluazuron | 73(5) | 102(5) | 77(1) | -26 | 76(12) | 87(4) | 81(16) | -35 | 78(9) | 72(15) | 73(11) | -53 | 78(14) | 83(2) | 77(10) | -19 | 70(1) | 96(13) | 85(14) | 25 |
| Chromafenozide | 97(6) | 89(5) | 72(10) | -6 | 93(5) | 106(3) | 100(6) | -23 | 97(4) | 82(2) | 84(13) | -11 | 92(3) | 101(2) | 72(2) | -7 | 89(2) | 77(6) | 90(7) | 10 |
| Clothianidin | 85(7) | 78(9) | 77(13) | 9 | 94(5) | 99(8) | 84(2) | -2 | 99(5) | 79(10) | 95(3) | 4 | 102(7) | 105(7) | 97(13) | 14 | 77(6) | 74(16) | 89(9) | 18 |
| Coumoxystrobin | 77(1) | 76(14) | 103(6) | -11 | 73(9) | 81(9) | 76(14) | -32 | 81(5) | 97(7) | 89(16) | -37 | 81(8) | 109(10) | 83(10) | -8 | 77(6) | 77(11) | 95(9) | 10 |
| Cyantraniliprole | 99(7) | 105(6) | 77(3) | -2 | 102(5) | 87(9) | 100(5) | -17 | 100(6) | 72(5) | 97(16) | -9 | 88(7) | 108(5) | 103(1) | 1 | 97(4) | 99(2) | 79(13) | 30 |
| Cyazofamid | 85(6) | 97(2) | 101(11) | -5 | 83(7) | 107(1) | 95(8) | -16 | 86(6) | 80(6) | 78(13) | -25 | 79(6) | 82(5) | 101(11) | -5 | 80(8) | 85(2) | 80(2) | 6 |
| CCIM (4-Chlor-2-cyano-5-(4-methylphenyl)imidazol) | 91(6) | 73(10) | 93(10) | 19 | 87(10) | 86(4) | 99(13) | 3 | 90(5) | 101(8) | 91(5) | 10 | 86(8) | 106(6) | 102(2) | 22 | 90(9) | 106(3) | 88(1) | 28 |
| Cyetpyrafen | 79(5) | 96(8) | 108(12) | -31 | 90(8) | 87(6) | 82(17) | -41 | 71(8) | 95(5) | 86(8) | -51 | 73(2) | 99(7) | 72(10) | -28 | 81(5) | 80(10) | 107(5) | 6 |
| Cyflufenamid | 85(7) | 98(8) | 80(6) | 1 | 79(7) | 100(10) | 87(6) | -17 | 88(5) | 83(7) | 81(9) | -14 | 85(4) | 87(1) | 104(12) | -6 | 84(5) | 74(14) | 88(1) | 7 |
| Cyflumetofen | 81(7) | 93(7) | 79(13) | -35 | 88(7) | 105(2) | 73(10) | -42 | 82(6) | 93(7) | 94(10) | -44 | 74(3) | 108(4) | 80(12) | -17 | 83(7) | 73(8) | 79(3) | 9 |
| Cymoxanil | 106(6) | 73(2) | 106(6) | -15 | 95(4) | 102(1) | 111(16) | -1 | 107(8) | 91(7) | 92(15) | 6 | 93(4) | 99(1) | 102(4) | 11 | 96(4) | 93(2) | 83(13) | 15 |
| Cyproconazole | 97(5) | 80(6) | 82(1) | 19 | 97(6) | 87(3) | 104(17) | -1 | 97(6) | 96(2) | 81(9) | 5 | 87(4) | 86(2) | 74(6) | 17 | 99(3) | 75(3) | 80(2) | 23 |
| Cyprodinil | 82(6) | 95(3) | 94(4) | -15 | 81(8) | 90(6) | 84(6) | 0 | 84(5) | 76(2) | 82(9) | 2 | 89(19) | 109(7) | 74(13) | -13 | 75(8) | 92(16) | 82(11) | 23 |
| Diclobutrazol | 91(6) | 85(4) | 72(7) | 10 | 93(6) | 106(3) | 86(12) | -15 | 94(4) | 88(14) | 87(4) | 3 | 89(4) | 109(5) | 92(10) | 15 | 90(5) | 75(12) | 100(8) | 22 |
| Diethofencarb | 99(6) | 96(13) | 84(9) | 6 | 93(7) | 109(2) | 78(2) | -3 | 100(6) | 81(7) | 100(5) | -3 | 92(5) | 77(3) | 76(7) | 12 | 90(3) | 84(2) | 83(12) | 15 |
| Difenoconazole | 84(4) | 89(6) | 79(8) | 4 | 85(6) | 88(4) | 75(9) | -14 | 97(6) | 73(3) | 99(10) | -8 | 87(9) | 102(4) | 74(5) | 4 | 102(16) | 112(16) | 77(12) | 16 |
| Diflubenzuron | 86(7) | 88(13) | 84(13) | -3 | 83(9) | 100(7) | 89(1) | -16 | 91(5) | 72(3) | 100(8) | -7 | 81(9) | 77(10) | 96(3) | 1 | 78(7) | 113(6) | 90(9) | 4 |
| Dimethomorph | 95(5) | 100(13) | 100(5) | -12 | 99(5) | 107(7) | 103(6) | -21 | 94(8) | 80(8) | 72(5) | -20 | 86(8) | 89(7) | 81(11) | 1 | 91(5) | 102(15) | 102(3) | 3 |
| Dimoxystrobin | 94(6) | 100(12) | 82(8) | 5 | 97(9) | 83(6) | 111(13) | -13 | 96(8) | 77(11) | 100(11) | -7 | 89(5) | 79(3) | 78(10) | 7 | 94(4) | 106(14) | 90(12) | 9 |
| Diniconazole | 93(8) | 74(7) | 102(1) | 9 | 100(7) | 94(4) | 101(4) | -9 | 99(6) | 77(2) | 91(6) | -2 | 85(3) | 94(10) | 112(3) | 11 | 93(5) | 111(15) | 107(6) | 20 |
| Emamectinbenzoate | 92(5) | 94(9) | 78(9) | -1 | 119(5) | 100(9) | 110(15) | -10 | 111(7) | 92(8) | 78(17) | -39 | 84(7) | 101(7) | 102(4) | 7 | 110(8) | 94(3) | 91(4) | -3 |
| Enestroburin | 83(7) | 107(4) | 73(6) | 1 | 88(7) | 87(1) | 87(5) | -16 | 87(7) | 83(16) | 79(5) | -13 | 77(6) | 101(3) | 109(5) | 1 | 84(9) | 100(7) | 95(7) | 18 |
| Epoxiconazole | 90(6) | 89(6) | 79(9) | 8 | 94(6) | 87(4) | 99(2) | -7 | 93(7) | 73(2) | 84(7) | 4 | 83(5) | 87(1) | 75(9) | 6 | 92(7) | 111(12) | 76(11) | 12 |
| Ethiprole | 99(5) | 99(1) | 73(10) | 17 | 106(5) | 104(8) | 94(15) | -22 | 100(7) | 100(6) | 73(12) | -16 | 89(4) | 88(10) | 72(3) | 2 | 99(3) | 109(15) | 94(7) | 11 |
| Ethirimol | 72(7) | 76(13) | 98(5) | 1 | 97(3) | 107(2) | 87(2) | -10 | 95(6) | 104(8) | 80(11) | -5 | 109(14) | 95(1) | 109(11) | 3 | 95(5) | 108(4) | 78(8) | 11 |
| Famoxadone | 74(9) | 89(9) | 84(10) | -5 | 77(10) | 105(6) | 109(17) | -18 | 76(7) | 88(7) | 76(4) | -18 | 77(3) | 110(2) | 99(13) | 4 | 79(10) | 113(16) | 77(12) | 10 |
| Fenamidone | 96(5) | 99(9) | 85(3) | 10 | 97(6) | 85(7) | 96(10) | -5 | 95(5) | 73(7) | 81(8) | -13 | 88(6) | 86(6) | 111(10) | 9 | 93(5) | 73(2) | 96(15) | 18 |
| Fenarimol | 92(7) | 89(13) | 111(2) | 19 | 90(8) | 83(6) | 100(12) | -19 | 92(6) | 83(9) | 76(6) | 2 | 85(5) | 87(6) | 88(5) | 17 | 88(5) | 99(5) | 77(4) | 20 |
| Fenbuconazole | 90(7) | 97(4) | 113(2) | 2 | 89(8) | 82(8) | 108(12) | -12 | 87(7) | 102(4) | 94(17) | -8 | 80(7) | 100(2) | 111(6) | 4 | 85(6) | 77(16) | 110(6) | 12 |
| Fenhexamid | 93(5) | 91(1) | 114(11) | 13 | 93(8) | 79(3) | 94(9) | 11 | 97(7) | 81(6) | 74(16) | 28 | 88(5) | 86(4) | 107(9) | 33 | 93(5) | 112(6) | 87(13) | 17 |
| Fenoxanil | 92(7) | 79(12) | 72(9) | -18 | 98(7) | 93(8) | 94(17) | -16 | 93(9) | 72(9) | 90(9) | -15 | 86(4) | 104(10) | 91(9) | -14 | 95(5) | 104(17) | 110(2) | 5 |
| Fenpropidin | 89(4) | 96(12) | 94(11) | 13 | 101(4) | 85(9) | 85(5) | -5 | 92(6) | 102(10) | 95(16) | 3 | 84(9) | 108(4) | 83(9) | 14 | 94(4) | 74(6) | 103(11) | 23 |
| Fenpropimorph | 91(4) | 92(9) | 103(12) | 1 | 92(6) | 101(8) | 109(14) | -13 | 99(5) | 95(10) | 90(15) | -6 | 88(4) | 107(4) | 107(6) | 2 | 90(2) | 109(17) | 88(13) | 13 |
| Fenpyrazamine | 95(6) | 92(13) | 83(9) | 8 | 93(6) | 78(9) | 110(16) | -5 | 95(6) | 96(14) | 97(16) | -4 | 88(4) | 99(2) | 85(5) | 0 | 94(4) | 97(9) | 79(15) | 24 |
| Fipronil | 93(5) | 89(13) | 74(4) | 20 | 94(7) | 91(10) | 92(5) | -2 | 95(6) | 72(5) | 95(14) | 0 | 88(3) | 104(6) | 88(10) | 15 | 89(5) | 81(10) | 90(5) | 31 |
| Fipronil-desulfinyl | 92(6) | 85(8) | 110(10) | 21 | 94(8) | 100(2) | 91(6) | -2 | 92(7) | 101(4) | 91(13) | 4 | 87(3) | 100(8) | 94(5) | 20 | 91(6) | 90(4) | 95(1) | 26 |
| Fipronil-sulfide | 89(6) | 71(5) | 80(4) | 6 | 89(8) | 85(2) | 101(3) | -15 | 89(5) | 73(10) | 80(8) | -10 | 83(2) | 104(1) | 75(12) | 5 | 85(6) | 110(7) | 78(9) | 18 |
| Fipronil-sulfone | 89(7) | 76(9) | 83(13) | -3 | 88(9) | 101(2) | 98(1) | -20 | 88(4) | 89(4) | 92(14) | -10 | 83(2) | 85(4) | 101(11) | 7 | 85(6) | 76(7) | 76(8) | 14 |
| Fluazinam | 91(4) | 73(1) | 111(6) | -1 | 75(2) | 110(6) | 94(2) | -30 | 74(6) | 86(5) | 91(17) | -33 | 96(8) | 105(2) | 90(3) | -2 | 72(8) | 73(10) | 78(1) | 17 |
| Flubendiamide | 93(6) | 87(3) | 113(12) | 55 | 106(3) | 77(10) | 80(16) | 8 | 90(8) | 93(11) | 98(11) | 11 | 86(4) | 89(8) | 80(7) | 49 | 96(3) | 109(15) | 98(11) | 36 |
| Fludioxonil | 82(7) | 97(10) | 111(12) | 13 | 86(7) | 84(1) | 84(16) | -5 | 87(7) | 81(11) | 83(7) | 6 | 95(13) | 105(4) | 80(4) | 9 | 87(5) | 110(4) | 80(11) | 11 |
| Flufenoxuron | 74(3) | 86(4) | 97(1) | -58 | 74(11) | 84(1) | 82(7) | -68 | 71(9) | 100(16) | 80(17) | -77 | 78(9) | 79(6) | 93(1) | -58 | 80(9) | 103(10) | 77(9) | -45 |
| Flumetralin | 80(1) | 105(13) | 89(10) | -10 | 73(3) | 84(1) | 110(6) | -32 | 71(7) | 90(15) | 92(11) | -36 | 80(2) | 87(2) | 106(7) | -9 | 76(11) | 104(10) | 109(7) | 12 |
| Flumorph | 99(7) | 87(13) | 84(4) | 6 | 95(4) | 99(8) | 88(1) | -8 | 97(6) | 89(2) | 85(5) | -2 | 89(6) | 94(10) | 105(5) | 8 | 92(3) | 90(9) | 107(14) | 20 |
| Fluopicolide | 91(4) | 77(5) | 101(3) | 4 | 89(7) | 99(7) | 86(10) | -12 | 97(6) | 79(5) | 72(8) | -3 | 89(6) | 80(7) | 82(2) | 1 | 87(4) | 87(17) | 105(14) | 7 |
| Fluopyram | 96(1) | 72(11) | 105(8) | 16 | 96(5) | 77(3) | 74(3) | -3 | 98(5) | 98(14) | 99(11) | 0 | 93(3) | 84(3) | 85(1) | 14 | 97(11) | 110(13) | 80(10) | 27 |
| Flusilazole | 88(6) | 74(12) | 112(1) | 12 | 94(6) | 103(1) | 89(9) | -5 | 99(5) | 86(9) | 99(17) | -1 | 85(5) | 91(1) | 113(1) | 13 | 94(5) | 104(4) | 95(15) | 19 |
| Flutolanil | 96(5) | 108(6) | 102(1) | 13 | 99(5) | 81(8) | 76(2) | -7 | 98(7) | 87(2) | 72(8) | 1 | 88(3) | 98(7) | 114(2) | 5 | 94(4) | 112(13) | 95(9) | 16 |
| Flutriafol | 100(7) | 90(11) | 94(11) | 19 | 99(4) | 80(10) | 109(3) | 0 | 98(7) | 96(5) | 96(6) | 7 | 93(5) | 79(4) | 82(12) | 16 | 97(5) | 111(5) | 85(14) | 24 |
| Fluxapyroxad | 95(6) | 100(3) | 100(3) | 0 | 89(5) | 106(2) | 97(16) | -15 | 96(6) | 94(2) | 86(4) | -4 | 88(4) | 108(4) | 83(1) | -6 | 88(3) | 93(9) | 88(14) | 4 |
| Forchlorfenuron | 90(7) | 83(10) | 72(5) | 13 | 91(7) | 99(3) | 100(16) | 1 | 89(6) | 72(16) | 99(16) | 9 | 81(10) | 80(6) | 111(12) | 16 | 88(6) | 94(3) | 109(14) | 20 |
| Hexaconazole | 88(6) | 103(6) | 87(2) | 17 | 95(6) | 106(10) | 103(7) | -4 | 96(7) | 94(16) | 89(15) | 4 | 89(5) | 100(9) | 80(11) | -3 | 94(4) | 83(16) | 85(1) | 21 |
| Hexaflumuron | 80(4) | 80(1) | 88(10) | 26 | 79(9) | 78(10) | 101(7) | -16 | 81(6) | 81(6) | 76(15) | -29 | 77(7) | 108(1) | 72(11) | 5 | 82(7) | 85(5) | 83(10) | 26 |
| Hexythiazox | 74(5) | 75(5) | 101(9) | 0 | 72(11) | 102(5) | 91(12) | -22 | 76(7) | 95(13) | 75(13) | -21 | 73(3) | 102(7) | 108(2) | 6 | 78(11) | 78(2) | 109(6) | 14 |
| Imazalil | 91(8) | 96(8) | 96(12) | -1 | 93(3) | 82(4) | 93(17) | -9 | 94(5) | 85(4) | 84(14) | -2 | 92(5) | 98(3) | 79(13) | 2 | 88(3) | 87(8) | 100(8) | 11 |
| Imidacloprid | 104(6) | 84(13) | 81(9) | 14 | 98(4) | 93(10) | 91(7) | 4 | 108(9) | 97(4) | 72(17) | 7 | 86(5) | 96(6) | 76(12) | 21 | 100(3) | 95(16) | 101(5) | 22 |
| Imidaclothiz | 106(6) | 76(10) | 113(12) | 13 | 86(5) | 101(4) | 108(14) | 2 | 95(7) | 87(15) | 92(17) | 5 | 90(7) | 88(8) | 74(11) | 13 | 85(3) | 85(6) | 79(10) | 19 |
| Indoxacarb | 90(5) | 86(15) | 82(5) | -19 | 80(10) | 104(6) | 96(5) | -36 | 81(7) | 81(16) | 96(10) | -35 | 72(5) | 77(5) | 75(12) | -18 | 88(9) | 92(17) | 90(4) | 4 |
| Ipconazole | 88(9) | 83(2) | 98(11) | 4 | 91(8) | 97(1) | 112(8) | -10 | 85(7) | 103(7) | 79(16) | 0 | 83(3) | 107(2) | 76(5) | 9 | 86(6) | 97(13) | 103(8) | 19 |
| Isoprothiolane | 95(6) | 92(14) | 114(11) | 1 | 94(5) | 110(5) | 85(11) | -17 | 95(5) | 96(8) | 83(12) | -7 | 85(5) | 97(1) | 101(11) | 0 | 94(3) | 73(11) | 81(4) | 15 |
| Isopyrazam | 88(5) | 106(6) | 77(10) | 5 | 87(6) | 101(7) | 81(8) | -13 | 86(6) | 83(12) | 99(6) | -7 | 83(4) | 103(6) | 98(3) | 4 | 88(3) | 83(9) | 103(12) | 15 |
| Ivermectin | 84(10) | 71(11) | 107(12) | -52 | 118(4) | 105(10) | 111(1) | -47 | 76(14) | 72(7) | 88(3) | -56 | 113(12) | 77(3) | 84(9) | -46 | 84(10) | 79(14) | 97(2) | -37 |
| Kresoxim-methyl | 87(6) | 108(8) | 98(9) | 12 | 86(6) | 96(4) | 79(13) | -8 | 74(6) | 83(5) | 88(11) | 24 | 79(10) | 83(1) | 106(12) | 39 | 83(5) | 97(6) | 105(1) | 14 |
| Lufenuron | 84(4) | 96(8) | 91(4) | -6 | 104(6) | 97(1) | 84(17) | -29 | 78(6) | 98(5) | 88(3) | -40 | 81(4) | 95(3) | 102(8) | -9 | 76(9) | 106(7) | 90(1) | 25 |
| Mandipropamid | 90(5) | 81(12) | 77(8) | -2 | 92(9) | 81(8) | 84(14) | -21 | 95(7) | 80(15) | 82(13) | -12 | 84(5) | 78(6) | 89(9) | -1 | 91(5) | 110(9) | 75(7) | 12 |
| Mepronil | 93(6) | 77(14) | 109(10) | 4 | 90(7) | 92(5) | 82(9) | -9 | 95(7) | 100(16) | 88(11) | -1 | 88(4) | 106(3) | 74(10) | 8 | 90(6) | 84(8) | 90(15) | 15 |
| Metaflumizone | 77(2) | 94(14) | 83(1) | 3 | 80(12) | 109(8) | 76(14) | -26 | 77(11) | 103(2) | 74(5) | -43 | 73(3) | 90(3) | 72(6) | 2 | 73(5) | 108(8) | 77(11) | 27 |
| Metalaxyl | 104(7) | 78(5) | 84(11) | 7 | 98(3) | 92(5) | 77(8) | -5 | 99(7) | 76(2) | 86(8) | 1 | 94(5) | 99(5) | 108(7) | 8 | 95(5) | 86(11) | 94(6) | 17 |
| Metconazole | 92(6) | 84(12) | 91(12) | 22 | 95(9) | 78(10) | 105(14) | 2 | 94(5) | 100(13) | 89(17) | 7 | 84(4) | 92(8) | 99(5) | 23 | 90(4) | 98(16) | 75(8) | 27 |
| Methoprene | 106(6) | 101(15) | 110(11) | -23 | 71(10) | 95(4) | 90(6) | -34 | 71(3) | 73(3) | 81(7) | -36 | 82(6) | 90(5) | 110(7) | -19 | 76(4) | 74(10) | 79(8) | -11 |
| Methoxyfenozide | 97(8) | 73(4) | 76(5) | 11 | 102(4) | 101(5) | 85(9) | -13 | 100(6) | 87(16) | 99(15) | -3 | 90(6) | 110(7) | 112(4) | -7 | 95(2) | 104(9) | 110(14) | 35 |
| Metrafenone | 86(8) | 92(14) | 100(10) | 2 | 84(8) | 90(3) | 103(8) | -15 | 85(5) | 80(11) | 80(3) | -9 | 84(6) | 86(6) | 75(9) | 0 | 82(6) | 106(10) | 97(1) | 14 |
| Myclobutanil | 95(8) | 108(7) | 79(9) | 16 | 94(5) | 106(3) | 76(16) | -3 | 99(7) | 87(10) | 76(3) | 6 | 88(4) | 95(7) | 108(1) | 17 | 94(3) | 86(11) | 80(14) | 21 |
| Novaluron | 86(5) | 104(12) | 107(5) | -22 | 87(10) | 109(1) | 110(12) | -41 | 95(7) | 96(12) | 92(13) | -47 | 74(4) | 104(10) | 80(9) | -24 | 86(6) | 105(16) | 85(14) | -8 |
| Oxadixyl | 103(8) | 104(9) | 105(7) | 2 | 87(6) | 107(3) | 92(11) | -24 | 102(10) | 96(12) | 76(10) | 0 | 97(5) | 103(7) | 103(3) | 1 | 95(4) | 96(8) | 79(12) | 0 |
| Paclobutrazol | 98(6) | 95(4) | 108(10) | 12 | 97(5) | 98(8) | 75(12) | -4 | 98(7) | 87(4) | 100(8) | 2 | 90(4) | 80(10) | 99(3) | 12 | 93(3) | 77(13) | 94(1) | 19 |
| Penconazole | 92(6) | 72(13) | 104(1) | -11 | 92(6) | 106(5) | 90(10) | -20 | 92(5) | 83(9) | 72(17) | -17 | 87(5) | 102(2) | 92(12) | -15 | 91(6) | 96(6) | 86(9) | -9 |
| Pencycuron | 86(6) | 100(10) | 106(10) | 6 | 85(10) | 100(7) | 79(5) | -12 | 85(5) | 72(10) | 77(4) | -7 | 81(5) | 85(6) | 73(12) | 6 | 84(8) | 98(14) | 81(1) | 15 |
| Penflufen | 94(5) | 73(2) | 89(5) | 10 | 96(6) | 109(7) | 76(11) | -8 | 95(6) | 85(12) | 88(13) | -2 | 87(3) | 110(8) | 112(2) | 11 | 94(2) | 89(3) | 105(8) | 17 |
| Penthiopyrad | 92(5) | 94(10) | 100(11) | 8 | 89(6) | 86(8) | 92(3) | -9 | 96(5) | 90(13) | 78(14) | 0 | 86(3) | 97(2) | 114(1) | 13 | 93(2) | 74(11) | 97(6) | 17 |
| Phenamacril | 101(6) | 105(8) | 100(1) | 4 | 95(4) | 84(3) | 109(7) | -4 | 98(7) | 84(10) | 80(9) | 2 | 92(5) | 92(9) | 81(12) | 6 | 92(3) | 109(3) | 109(2) | 13 |
| Phoxim | 78(6) | 97(1) | 87(4) | -5 | 76(8) | 96(5) | 88(16) | -13 | 74(3) | 96(15) | 87(7) | -9 | 71(8) | 84(1) | 104(7) | -6 | 72(8) | 111(16) | 86(7) | 7 |
| Picoxystrobin | 89(5) | 75(1) | 104(7) | -7 | 94(8) | 81(3) | 102(17) | -14 | 91(6) | 87(11) | 79(10) | -15 | 85(3) | 78(10) | 72(3) | -2 | 91(6) | 87(2) | 97(14) | 6 |
| Piperonylbutoxide | 80(5) | 90(13) | 85(4) | -11 | 80(8) | 81(10) | 112(11) | -21 | 83(6) | 98(4) | 80(5) | -17 | 80(7) | 84(2) | 94(3) | -11 | 78(6) | 80(16) | 78(2) | 9 |
| Prochloraz | 86(7) | 79(10) | 98(5) | 7 | 88(7) | 94(4) | 111(13) | -5 | 88(6) | 91(4) | 92(17) | -4 | 82(6) | 99(9) | 111(2) | 6 | 89(13) | 81(6) | 79(12) | 14 |
| Prochlorazmetabolitebts44595 | 93(5) | 77(6) | 77(8) | 7 | 90(7) | 102(5) | 109(4) | -11 | 94(6) | 72(14) | 79(7) | -18 | 87(4) | 78(9) | 114(2) | -14 | 91(8) | 83(11) | 82(5) | 13 |
| Prochlorazmetabolitebts44596 | 91(7) | 103(3) | 92(6) | 34 | 87(8) | 95(1) | 112(9) | 13 | 91(6) | 84(14) | 88(9) | 12 | 86(5) | 78(6) | 74(8) | 24 | 92(11) | 102(8) | 75(12) | 26 |
| Propiconazole | 89(7) | 89(6) | 112(13) | 9 | 92(9) | 87(8) | 93(16) | -24 | 100(13) | 85(2) | 94(12) | -4 | 89(8) | 80(1) | 75(5) | 7 | 86(6) | 78(11) | 95(6) | 15 |
| Pyraclostrobin | 81(2) | 106(15) | 107(13) | 9 | 73(5) | 107(1) | 110(15) | -8 | 73(7) | 93(13) | 92(16) | -2 | 84(2) | 100(9) | 78(8) | 12 | 75(10) | 112(15) | 96(2) | 18 |
| Pyrametostrobin | 86(7) | 88(2) | 83(9) | 1 | 82(7) | 91(1) | 103(17) | -15 | 86(5) | 76(15) | 97(12) | -11 | 78(10) | 86(9) | 86(7) | 2 | 82(5) | 92(14) | 109(14) | 13 |
| Pyraoxystrobin | 78(6) | 80(7) | 110(7) | 7 | 74(7) | 78(10) | 99(16) | -16 | 78(7) | 101(10) | 91(13) | -9 | 75(14) | 83(8) | 104(7) | 9 | 74(6) | 102(13) | 82(5) | 15 |
| Pyrethrini | 78(10) | 76(8) | 76(9) | -8 | 79(11) | 83(3) | 86(14) | -29 | 78(5) | 94(4) | 81(6) | -32 | 71(8) | 93(3) | 110(2) | -3 | 75(10) | 102(6) | 108(7) | 5 |
| Pyrethrinii | 90(1) | 84(11) | 113(13) | 5 | 88(13) | 100(8) | 106(10) | -20 | 84(6) | 89(7) | 88(6) | -13 | 77(15) | 96(9) | 113(5) | 5 | 83(11) | 103(5) | 81(6) | 12 |
| Pyridaben | 76(7) | 102(4) | 110(5) | -16 | 73(9) | 81(6) | 100(15) | -34 | 71(6) | 92(16) | 96(9) | -45 | 74(7) | 99(8) | 89(12) | -13 | 73(8) | 104(7) | 77(13) | 9 |
| Pyrimethanil | 90(6) | 79(6) | 108(1) | -13 | 93(6) | 102(4) | 93(12) | -23 | 100(8) | 73(2) | 77(13) | -17 | 104(9) | 81(5) | 90(9) | -12 | 86(6) | 82(12) | 90(9) | 14 |
| Pyrimorph | 89(6) | 74(12) | 104(9) | 4 | 94(7) | 77(9) | 96(4) | -12 | 93(7) | 95(16) | 77(10) | -5 | 84(6) | 81(1) | 108(9) | 8 | 90(5) | 104(10) | 105(10) | 18 |
| Pyrisoxazole | 90(7) | 93(11) | 83(10) | -4 | 90(5) | 110(9) | 109(12) | -6 | 89(7) | 76(13) | 97(17) | 9 | 102(18) | 104(4) | 76(6) | 9 | 86(5) | 105(5) | 84(7) | 20 |
| Sedaxane | 95(6) | 98(6) | 72(10) | 12 | 94(6) | 82(10) | 85(15) | -7 | 94(7) | 73(14) | 76(15) | -3 | 87(5) | 82(6) | 77(6) | 10 | 91(4) | 109(11) | 82(7) | 17 |
| Silthiofam | 95(6) | 102(7) | 106(8) | 10 | 96(7) | 94(5) | 111(5) | -5 | 98(6) | 100(5) | 82(6) | 2 | 89(4) | 79(6) | 92(5) | 10 | 94(4) | 107(10) | 81(11) | 19 |
| Spinetoramj | 85(8) | 86(8) | 95(4) | -22 | 83(6) | 109(2) | 75(10) | -43 | 97(6) | 89(10) | 85(17) | -38 | 84(3) | 89(8) | 90(1) | -10 | 83(4) | 107(15) | 107(8) | -32 |
| Spinetoraml | 83(6) | 98(4) | 74(5) | -42 | 112(6) | 109(4) | 84(1) | -28 | 75(5) | 83(6) | 98(5) | -53 | 81(6) | 110(7) | 102(13) | -33 | 77(4) | 111(9) | 95(5) | -26 |
| N-demethyl-175-J | 98(6) | 77(7) | 109(1) | 105 | 100(6) | 99(2) | 82(7) | 50 | 103(5) | 82(5) | 87(3) | 34 | 87(5) | 84(2) | 82(8) | 103 | 86(8) | 88(14) | 102(11) | 91 |
| N-formyl-175-J | 95(5) | 103(13) | 94(2) | 13 | 84(5) | 105(5) | 74(1) | -19 | 88(6) | 99(12) | 96(8) | -10 | 81(8) | 108(4) | 111(10) | 9 | 83(3) | 81(15) | 79(4) | 17 |
| Spinosad A | 76(2) | 103(7) | 107(11) | -12 | 101(11) | 80(3) | 88(14) | -21 | 93(9) | 80(10) | 83(11) | -38 | 81(8) | 99(6) | 104(2) | -3 | 91(5) | 82(5) | 91(8) | -3 |
| Spinosad D | 80(7) | 82(14) | 109(8) | -4 | 105(7) | 101(10) | 103(6) | -13 | 93(7) | 104(16) | 95(12) | -37 | 83(1) | 104(9) | 108(12) | -5 | 85(3) | 101(14) | 110(8) | -4 |
| Spirodiclofen | 81(8) | 107(3) | 93(2) | -7 | 81(11) | 105(6) | 92(1) | -32 | 75(8) | 90(11) | 86(17) | -37 | 78(4) | 88(6) | 112(9) | -3 | 79(9) | 92(3) | 103(15) | 15 |
| Spiromesifen | 73(5) | 101(9) | 79(6) | 5 | 108(15) | 108(10) | 85(7) | -20 | 79(8) | 89(16) | 92(17) | -19 | 84(13) | 107(10) | 100(9) | 8 | 88(7) | 101(8) | 76(6) | 9 |
| Spirotetramat | 97(6) | 82(11) | 80(2) | 24 | 92(4) | 79(8) | 112(13) | 6 | 98(6) | 103(10) | 93(10) | 12 | 101(12) | 104(4) | 108(7) | 30 | 93(3) | 80(2) | 91(14) | 20 |
| Spirotetramat-enol | 104(6) | 96(12) | 85(7) | 18 | 81(6) | 107(3) | 77(10) | -6 | 88(6) | 98(12) | 96(6) | -6 | 110(15) | 106(7) | 104(3) | 18 | 97(5) | 82(6) | 83(7) | 30 |
| Spirotetramat-keto-hydroxy | 101(6) | 106(2) | 82(7) | 11 | 101(3) | 105(7) | 79(15) | -6 | 104(7) | 83(10) | 95(13) | 3 | 96(8) | 107(1) | 107(12) | 13 | 98(4) | 81(9) | 102(3) | 29 |
| Spirotetramat-mono-hydroxy | 102(7) | 92(9) | 101(6) | -1 | 99(4) | 108(4) | 81(12) | -11 | 112(5) | 76(12) | 73(8) | -6 | 93(4) | 100(1) | 73(11) | 6 | 96(6) | 104(7) | 82(3) | 26 |
| Sulfoxaflor | 108(5) | 74(10) | 87(4) | 3 | 87(5) | 107(5) | 109(5) | 6 | 100(4) | 72(6) | 76(14) | 12 | 89(6) | 94(3) | 77(2) | 5 | 98(6) | 76(15) | 89(9) | 30 |
| Tebuconazole | 95(5) | 92(1) | 80(4) | -11 | 77(6) | 109(2) | 106(10) | -30 | 93(6) | 72(16) | 81(12) | -12 | 85(3) | 86(1) | 74(6) | -15 | 89(5) | 96(13) | 75(15) | 2 |
| Tebufenozide | 93(6) | 103(15) | 93(9) | 14 | 98(5) | 98(10) | 85(13) | -8 | 101(6) | 77(6) | 86(6) | -6 | 86(3) | 102(6) | 108(6) | 8 | 90(2) | 96(17) | 92(14) | 13 |
| Teflubenzuron | 79(10) | 91(9) | 86(13) | 44 | 77(9) | 96(1) | 78(6) | 6 | 91(6) | 92(10) | 91(11) | 18 | 76(9) | 102(9) | 80(1) | 42 | 81(8) | 86(14) | 102(14) | 37 |
| Tetrachlorantraniliprole | 94(9) | 91(15) | 83(11) | -21 | 102(10) | 94(5) | 96(2) | -40 | 97(7) | 87(7) | 93(12) | -32 | 83(4) | 102(7) | 96(7) | 2 | 98(6) | 87(5) | 84(13) | 2 |
| Tetraconazole | 91(6) | 74(13) | 101(5) | 12 | 94(5) | 96(9) | 93(10) | -10 | 95(6) | 99(13) | 74(7) | 0 | 87(2) | 98(2) | 111(10) | 13 | 92(4) | 98(16) | 90(5) | 19 |
| Thiabendazole | 90(8) | 106(10) | 79(8) | -3 | 102(6) | 79(5) | 106(1) | -11 | 85(7) | 79(6) | 73(14) | -3 | 83(4) | 89(6) | 85(4) | -4 | 79(3) | 86(11) | 79(8) | 10 |
| Thiacloprid | 94(5) | 89(3) | 81(9) | 1 | 89(5) | 104(8) | 107(2) | -6 | 97(6) | 85(10) | 80(14) | -1 | 89(5) | 92(7) | 73(5) | 3 | 91(1) | 84(12) | 92(5) | 13 |
| Thiamethoxam | 72(8) | 80(10) | 76(5) | -5 | 96(3) | 101(6) | 78(2) | -13 | 106(15) | 75(2) | 79(4) | -6 | 83(18) | 92(8) | 106(5) | 1 | 91(7) | 90(3) | 95(8) | 5 |
| Thidiazuron | 91(9) | 71(15) | 96(1) | 23 | 86(6) | 94(10) | 96(11) | 3 | 93(8) | 100(5) | 92(16) | 10 | 80(16) | 107(7) | 95(4) | 33 | 86(6) | 106(9) | 82(11) | 20 |
| Thifluzamide | 89(6) | 108(5) | 97(13) | 0 | 110(6) | 88(2) | 87(12) | -22 | 98(8) | 75(13) | 94(17) | -6 | 89(4) | 86(1) | 95(13) | 22 | 98(4) | 81(12) | 88(12) | 25 |
| Triadimefon | 99(6) | 71(13) | 100(7) | 16 | 102(7) | 81(8) | 104(9) | -2 | 97(6) | 82(6) | 74(17) | 4 | 90(5) | 107(5) | 73(7) | 16 | 98(5) | 90(7) | 82(12) | 19 |
| Triadimenol | 96(4) | 72(7) | 80(12) | 11 | 94(5) | 103(6) | 105(14) | 6 | 98(6) | 99(7) | 81(15) | -11 | 84(2) | 78(9) | 84(12) | -8 | 98(5) | 95(12) | 102(3) | 20 |
| Tricyclazole | 95(7) | 99(2) | 86(9) | 1 | 90(4) | 109(7) | 80(3) | -4 | 91(6) | 77(16) | 88(6) | 4 | 87(7) | 97(4) | 77(2) | 3 | 86(2) | 93(10) | 95(11) | 15 |
| Trifloxystrobin | 85(7) | 85(4) | 106(11) | 11 | 87(7) | 104(1) | 73(12) | -16 | 90(3) | 79(11) | 100(16) | -8 | 77(7) | 107(4) | 86(11) | 11 | 100(16) | 85(6) | 105(13) | 25 |
| Triflumizole | 81(9) | 94(5) | 82(7) | 15 | 91(7) | 99(8) | 79(8) | -7 | 85(8) | 78(7) | 95(12) | -2 | 90(4) | 86(1) | 78(13) | 3 | 84(3) | 82(12) | 103(14) | 17 |
| Triflumizolemetabolite  FM-6-1Ethanimidamide | 73(9) | 102(5) | 107(1) | -9 | 94(3) | 96(4) | 77(7) | -14 | 99(5) | 97(4) | 79(5) | -4 | 99(10) | 102(6) | 74(11) | 2 | 95(4) | 94(17) | 92(14) | 6 |
| Triflumuron | 83(8) | 103(14) | 110(13) | -9 | 82(8) | 99(3) | 80(14) | -13 | 86(4) | 83(10) | 98(9) | -18 | 78(9) | 105(10) | 109(6) | -3 | 78(9) | 84(9) | 108(13) | 19 |
| Triticonazole | 97(6) | 85(9) | 76(7) | 24 | 92(6) | 104(1) | 98(17) | -10 | 97(6) | 90(7) | 90(17) | -9 | 89(3) | 94(6) | 79(12) | 5 | 95(4) | 80(2) | 83(7) | 15 |
| Uniconazole | 95(7) | 74(2) | 98(4) | 18 | 99(4) | 80(7) | 84(9) | -3 | 96(8) | 83(7) | 73(15) | 4 | 89(3) | 85(5) | 110(3) | 18 | 95(4) | 95(9) | 110(12) | 21 |
| Zoxamide | 87(6) | 104(12) | 105(6) | 11 | 89(9) | 94(7) | 111(6) | -6 | 92(5) | 88(2) | 74(8) | -1 | 85(5) | 91(7) | 94(12) | 14 | 86(7) | 77(7) | 92(14) | 20 |
